# Secretory MPP3 reinforce myeloid differentiation trajectory and amplify myeloid cell production

**DOI:** 10.1084/jem.20230088

**Published:** 2023-04-26

**Authors:** Yoon-A Kang, Hyojung Paik, Si Yi Zhang, Jonathan J. Chen, Oakley C. Olson, Carl A. Mitchell, Amelie Collins, James W. Swann, Matthew R. Warr, Rong Fan, Emmanuelle Passegué

**Affiliations:** 1 Columbia Stem Cell Initiative, Department of Genetics and Development, Columbia University, New York, NY, USA; 2 Eli and Edythe Broad Center of Regeneration Medicine and Stem Cell Research, Department of Medicine, Hem/Onc Division, University of California, San Francisco, San Francisco, CA, USA; 3 Center for Applied Scientific Computing, Korea Institute of Science and Technology Information, and University of Science and Technology, Daejeon, South Korea; 4 Department of Biomedical Engineering, Yale University, New Haven, CT, USA

## Abstract

Hematopoietic stem cells (HSC) and downstream lineage-biased multipotent progenitors (MPP) tailor blood production and control myelopoiesis on demand. Recent lineage tracing analyses revealed MPPs to be major functional contributors to steady-state hematopoiesis. However, we still lack a precise resolution of myeloid differentiation trajectories and cellular heterogeneity in the MPP compartment. Here, we found that myeloid-biased MPP3 are functionally and molecularly heterogeneous, with a distinct subset of myeloid-primed secretory cells with high endoplasmic reticulum (ER) volume and FcγR expression. We show that FcγR^+^/ER^high^ MPP3 are a transitional population serving as a reservoir for rapid production of granulocyte/macrophage progenitors (GMP), which directly amplify myelopoiesis through inflammation-triggered secretion of cytokines in the local bone marrow (BM) microenvironment. Our results identify a novel regulatory function for a secretory MPP3 subset that controls myeloid differentiation through lineage-priming and cytokine production and acts as a self-reinforcing amplification compartment in inflammatory stress and disease conditions.

## Teaser

Kang et al. identify a novel secretory subset of myeloid-biased multipotent progenitors, MPP3, which enforces myeloid lineage differentiation through lineage-priming and cytokine secretion and serves an essential regulatory function to amplify myeloid cell production in stress and disease conditions.

## Graphical Abstract

**Figure:**
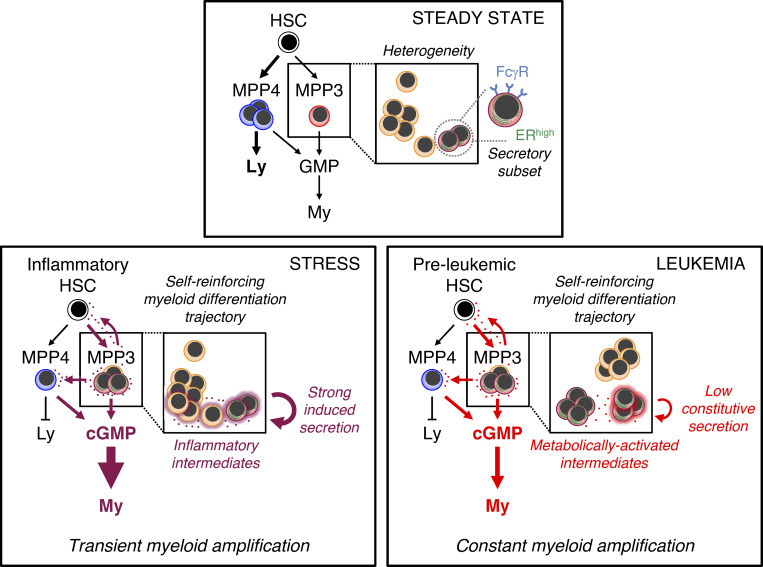

## Introduction

Myelopoiesis is a demand-adapted process where hematopoietic stem cells (HSC) and a collection of progenitor cells integrate signals from their environment and tailor the output of the myeloid lineage to meet the specific needs of the organism and respond to physiological challenges (Yamashita et al., 2020). Emergency myelopoiesis is induced to amplify myeloid cell production either acutely in stress conditions or constitutively in various disease contexts, resulting in a major reorganization of the hematopoietic stem and progenitor cell (HSPC) compartment at the top of the hematopoietic hierarchy (Olson et al., 2020). Quiescent HSCs are first activated, leading to expansion of myeloid-biased MPP2 and MPP3, and to myeloid reprogramming of lymphoid-biased MPP4, resulting in the formation of self-renewing granulocyte/macrophage progenitor (GMP) patches and their expansion into GMP clusters that drive local burst production of mature myeloid cells in the bone marrow (BM) microenvironment (Reynaud et al., 2011; Pietras et al., 2015; Hérault et al., 2017). The remodeling of the multipotent progenitor (MPP) compartment is triggered in part by low Notch and high Wnt activity in HSCs (Kang et al., 2020), and by proinflammatory cytokines like IL-6, IL-1, or TNFα that stimulate many steps of this regenerative program to amplify myeloid cell production (Reynaud et al., 2011; Pietras et al., 2016; Hérault et al., 2017; Yamashita and Passegué, 2019). Interestingly, HSPCs themselves secrete cytokines upon inflammatory stimuli (Zhao et al., 2014), raising the intriguing possibility that autocrine or paracrine signaling in the local BM niche might play an important regulatory function in controlling emergency myelopoiesis.

Single-cell RNA-sequencing (scRNA-seq) analyses and barcoding lineage tracing strategies have considerably advanced our knowledge of lineage specification and revealed molecular heterogeneity as well as mixed lineage expression patterns in distinct HSPC populations (Dahlin et al., 2018; Giladi et al., 2018; Rodriguez-Fraticelli et al., 2020; Wilson et al., 2015). It is also becoming apparent that lineage priming in multipotent MPPs has a significant functional impact on the type of mature blood cell being produced (Weinreb et al., 2020) and that localized microdomains in the BM niche play essential roles in controlling myeloid cell differentiation (Hérault et al., 2017; Zhang et al., 2021). However, we still lack a clear understanding of the regulatory mechanisms enforcing lineage trajectory choice and myeloid commitment at the top of the hematopoietic hierarchy and the precise role of myeloid-biased MPPs in amplifying myeloid cell production. This is particularly important given the major functional role recently uncovered for the MPP compartment in controlling blood production in native conditions (Sun et al., 2014; Busch et al., 2015; Rodriguez-Fraticelli et al., 2018). Here, we show that a myeloid-primed secretory subset of MPP3 is an important self-reinforcing amplification compartment controlling myeloid differentiation in stress and disease conditions, acting through both lineage-priming and differential cytokine production with paracrine/autocrine effects in the local BM niche.

## Results

### Enhanced cytokine secretion capacity of MPP3

**Figure S1.**
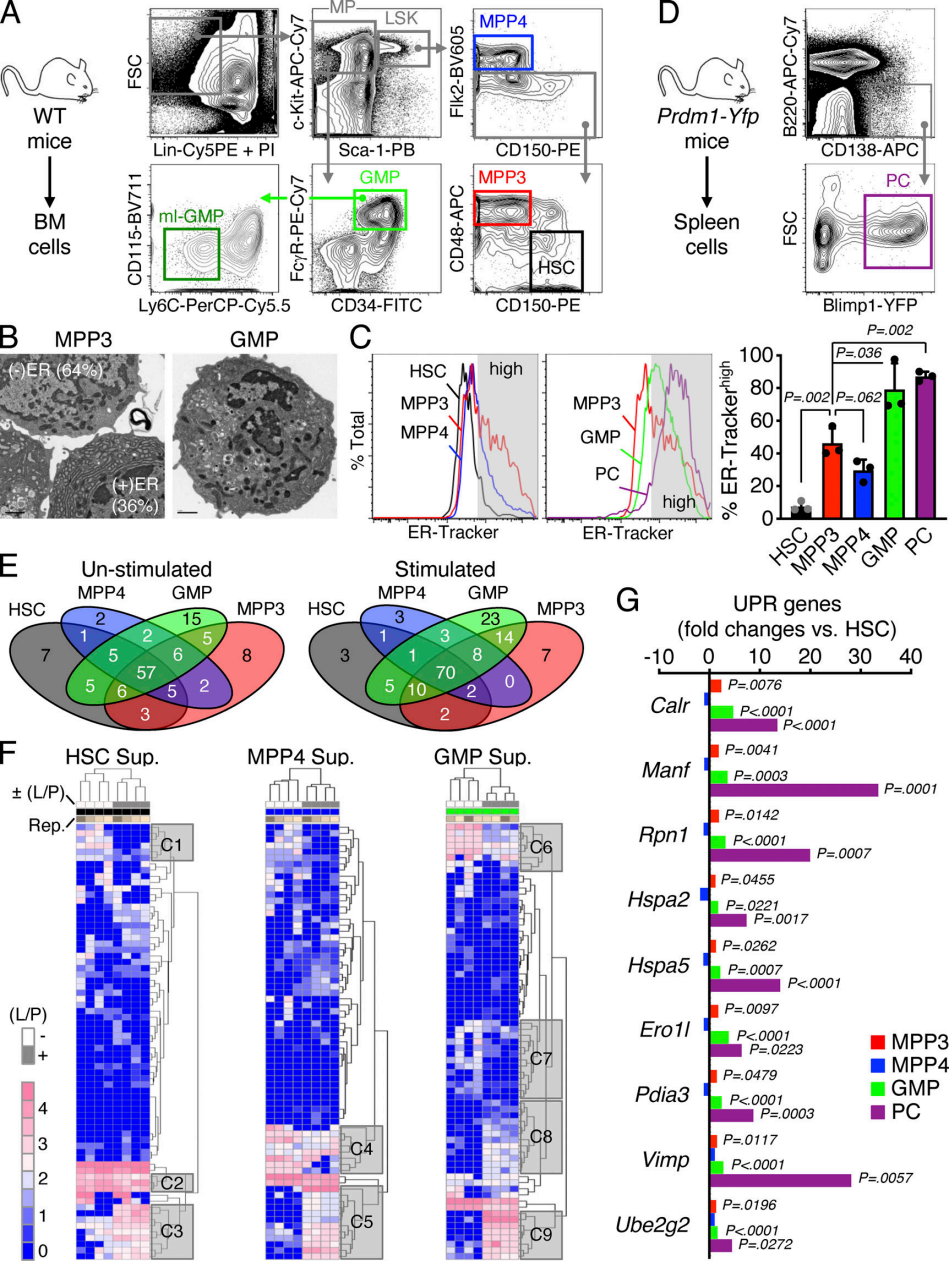
**Secretory activity of HSPCs. (A)** Gating strategy used for identifying and isolating BM HSCs and MPPs (MPP3 and MPP4) from the Lin^−^/Sca-1^+^/c-Kit^+^ (LSK) HSPC compartment, as well as GMP and ml-GMP subsets from the Lin^−^/Sca-1^−^/c-Kit^+^ myeloid progenitor (MP) compartment in WT donor mice. **(B)** Representative example of TEM images used to quantify the percentage of MPP3 with high (+) and low (−) ER volume (*n* = 69 cells total). Representative TEM image of GMP is shown for comparison to illustrate the differences in morphology. **(C)** ER-Tracker staining of HSPCs, GMPs, and plasma cells (PC) with representative FACS plots and quantification of ER-Tracker^high^ fraction (gray shaded area on histograms). Data are means ± SD (three independent experiments), and significance was assessed by a two-tailed unpaired Student’s *t* test. **(D)** Gating strategy used for identifying and isolating splenic plasma cells from *Prdm1-Yfp* mice. **(E and F)** Secretory activity of HSPCs and GMPs with (E) overlap in secreted cytokines between populations, and (F) heatmap of unsupervised clustering of secreted cytokines after quantile normalization. Supernatants (Sup.) were collected upon culture of 10,000 cells for 24 h in 150 µl base media ± LPS/Pam3CSK4 (L/P) stimulation; Rep., independent repeats. Uniquely secreted cytokines by each population and representative clusters (C1 to C9) of secreted cytokines changed upon stimulation are provided in Table S1. **(G)** SABiosciences PCR array of UPR genes in HSPCs, GMPs, and plasma cells (*n* = 3). Results are expressed as log_2_ mean fold expression relative to HSCs (set to 0). Significance was assessed by a two-tailed unpaired Student’s *t* test.

**Figure 1.**
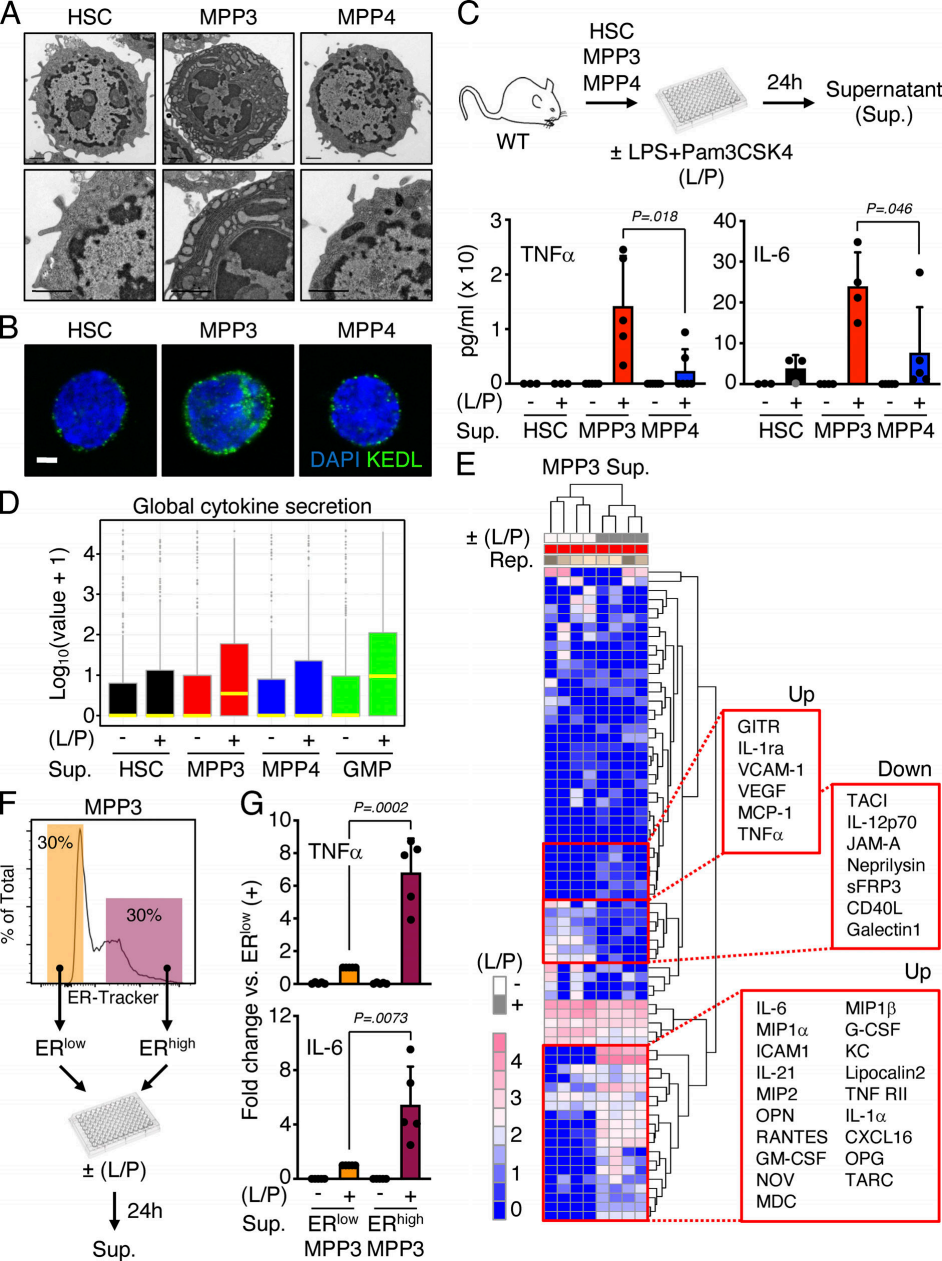
**Secretory features of MPP3. (A)** Representative TEM images of HSC, MPP3, and MPP4. Scale bar, 1 μm. **(B)** Representative immunofluorescence images of HSC, MPP3, and MPP4 stained for the ER marker KEDL. Scale bar, 3 μm. **(C)** Differential secretion of TNFα and IL-6 by HSC, MPP3, and MPP4 upon stimulation. Experimental schemes and results from ELISA measurements are shown (three independent experiments). Supernatants were collected upon culture of 10,000 cells for 24 h in 150 µl base media ± LPS/Pam3CSK4 (L/P) stimulation. **(D and E)** Stimulated MPP3 are the most secretory HSPCs with (D) box plots of secreted cytokine intensity by HSC, MPP3, and MPP4 upon stimulation (yellow lines represent mean values), and (E) heatmap of unsupervised clustering of MPP3-secreted cytokines after quantile normalization (representative increased [up] and decreased [down] cytokines upon stimulation are indicated on the right). Results are from 24-h culture supernatants analyzed with the Raybiotech 200 mouse cytokine array (four independent experiments). **(F)** Experimental scheme for isolating and analyzing ER^high^ (top 30% of ER-Tracker staining) and ER^low^ (bottom 30% of ER-Tracker staining) MPP3 subsets. **(G)** Differential secretion of IL-6 and TNFα by ER^high^ vs. ER^low^ MPP3 upon stimulation. Results are from 24-h culture supernatants analyzed by Luminex cytokine bead array (two independent experiments). Data are means ± SD except when indicated, and significance was assessed by a two-tailed unpaired Student’s *t* test.

**Figure S2.**
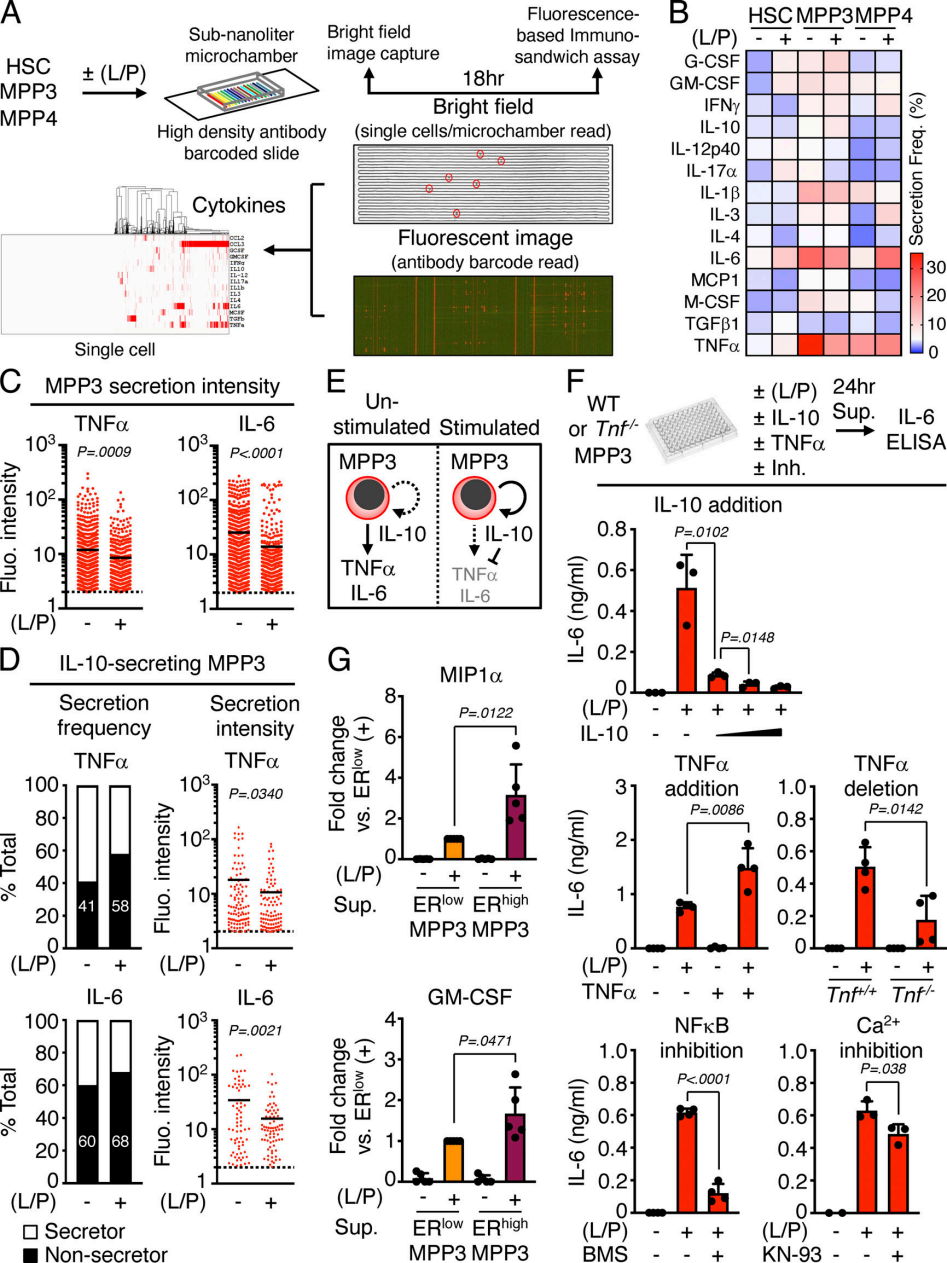
**Autocrine effect of MPP3 secretion. (A–D)** Secretory activity of HSPCs at the single cell level with (A) experimental scheme of HSC, MPP3, and MPP4 single-cell secretion assay ± LPS/Pam3CSK4 (L/P) stimulation for 18 h in culture (14 known cytokines were preselected for this assay); (B) heatmap of secretion frequency by single unstimulated/stimulated HSC, MPP3, and MPP4; (C) TNFα and IL-6 secretion intensity by all unstimulated/stimulated secreting MPP3. Cells with fluorescence (Fluo.) signal intensity above threshold (set to 2) are counted as secretors, and black lines represent mean values; and (D) TNFα and IL-6 secretion frequency and secretion intensity by IL-10–secreting MPP3. Data are from four independent experiments. **(E)** Model depicting the effect of IL-10 on TNFα and IL-6 secretion by individual MPP3. **(F)** Changes in IL-6 secretion by MPP3 upon IL-10 addition (10, 30, or 100 ng/ml), TNFα addition (1 µg/ml), TNFα genetic deletion, and NF-κB (BMS345541, 2 µM) or Ca^2+^ (KN-93, 2 µM) signaling inhibition. Supernatants were collected upon the culture of 10,000 WT or *Tnf*^−*/*−^ MPP3 for 24 h in 150 µl base media or full cytokine media (for TNFα addition) ± LPS/Pam3CSK4 (L/P) stimulation and the indicated inhibitor. Results are ELISA measurements (two independent experiments). **(G)** Differential secretion of MIP1α and GM-CSF by ER^high^ vs. ER^low^ MPP3 upon stimulation. Results are Luminex cytokine bead array measurement of 24-h supernatants (two independent experiments). Data are means ± SD except when indicated, and significance was assessed by a two-tailed unpaired Student’s *t* test.

To characterize HSPC secretory activity, we focused on several well-defined phenotypic populations including HSCs (Lin^−^/c-Kit^+^/Sca-1^+^/Flk2^−^/CD48^−^/CD150^+^), myeloid-biased MPP3 (Lin^−^/c-Kit^+^/Sca-1^+^/Flk2^−^/CD48^+^/CD150^−^), and lymphoid-biased MPP4 (Lin^−^/c-Kit^+^/Sca-1^+^/Flk2^+^), which we compared to myeloid-committed GMPs (Lin^−^/c-Kit^+^/Sca-1^−^/FcγR^+^/CD34^+^; Fig. S1 A). Strikingly, we found that approximately one-third of MPP3 had high ER volume by transmission electron microscopy (TEM; Fig. 1 A and Fig. S1 B). We confirmed the presence of an ER^high^ subset of MPP3 by immunofluorescence microscopy with the ER marker KDEL and flow cytometry using the ER-Tracker dye with ∼31.0 ± 10.4% (*n* = 20) of the MPP3 compartment identified as ER^high^ MPP3 at steady state (Fig. 1 B and Fig. S1 C). Interestingly, the dense rough ER structure observed in a subset of MPP3 was morphologically distinct from GMPs but was similar to the secretory apparatus found in specialized immunoglobulin-producing plasma cells (Fawcett, 1966), although MPP3 expressed different surface markers from plasma cells (Fig. S1, C and D). We then directly tested the secretory activity of MPP3 by treating isolated HSPC populations with a previously described inflammatory LPS/Pam3CSK4 stimulus (Zhao et al., 2014) before collecting supernatants 24 h later for secretome analyses. Stimulated MPP3 secreted higher levels of TNFα and IL-6 than HSCs and MPP4, as shown by ELISA, and displayed a global increase in cytokine secretion as measured with the Raybiotech 200 mouse cytokine array, which was much higher than other HSPC populations and in the range of stimulated GMPs (Fig. 1, C and D). Detailed examination of each HSPC population and GMPs revealed complex cell type–specific secretory patterns with a set of unique cytokines secreted per population with or without (±) stimulation and shared cytokines secreted at various levels by each population (Fig. 1 E; Fig. S1, E and F; and Table S1). In this context, stimulated MPP3 showed increased secretion of many pro-inflammatory and pro-myeloid differentiation cytokines including IL-1α, G-CSF, and GM-CSF, and decreased production of regulatory factors controlling immune cell function like TACI or CD40L (Fig. 1 E). Consistently, MPP3 expressed unfolded protein response (UPR) genes in the range of GMPs but to a lower extent than specialized secretory cells like plasma cells (Fig. S1 G). We also investigated MPP3 secretion at the single-cell level using a set of 14 preselected cytokines and an established nanofluidic technology (Fig. S2 A; Chen et al., 2019). We confirmed the higher overall secretory activity of MPP3 compared with HSCs and MPP4 (Fig. S2 B). However, at the single-cell level, stimulated MPP3 secreted less TNFα and IL-6 than unstimulated MPP3, possibly due to their concomitant production of IL-10, which is a known suppressor of IL-6 and TNFα production (Fig. S2, C–E; de Waal Malefyt et al., 1991). In fact, the direct addition of IL-10 to bulk MPP3 culture suppressed IL-6 secretion in a dose-dependent manner (Fig. S2 F). This suppressive effect of IL-10 was probably overcome in bulk culture due to the strong pro-secretion effect of other cytokines like TNFα itself (Fig. S2 F). We also confirmed a role for the classical mechanisms regulating cellular secretion (Anantharam and Kreutzberger, 2019; Liu et al., 2017), with inhibition of NF-κB and Ca^2+^-dependent signaling impairing IL-6 secretion from stimulated MPP3 (Fig. S2 F). To directly separate high from low secretory MPP3, we took advantage of their difference in ER volume and subfractionated MPP3 into ER^high^ (top 30%) and ER^low^ (bottom 30%) subsets based on ER-Tracker staining (Fig. 1 F). We confirmed significantly higher secretion of many cytokines, including TNFα and IL-6, in stimulated ER^high^ MPP3 (Fig. 1 G and Fig. S2 G). Taken together, these results demonstrate that MPP3 are the most secretory HSPCs, identify a unique ER^high^ MPP3 subset that robustly produces many proinflammatory/myeloid differentiation cytokines upon inflammatory stimulus, and suggest an autocrine effect of MPP3 secretion.

### MPP3 have distinct molecular subsets

**Figure 2.**
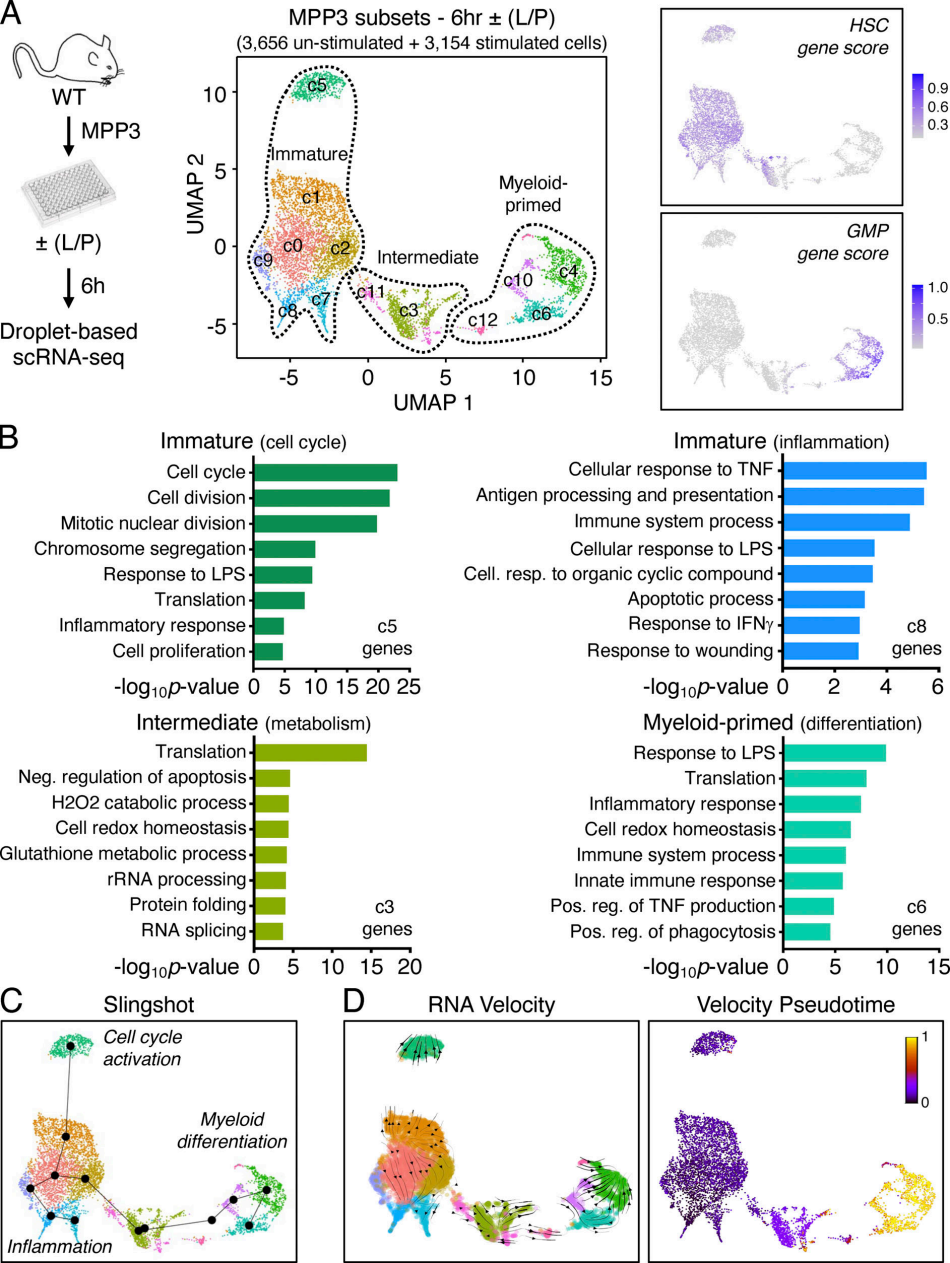
**MPP3 molecular heterogeneity and inflammatory remodeling. (A)** UMAP representation of unstimulated/stimulated MPP3 scRNA-seq dataset with experimental scheme (left) and gene module scoring for HSC and GMP genes (right). Results are from isolated MPP3 cultured for 6 h ± LPS and Pam3CSK4 (L/P) stimulation. **(B)** GO analysis of the indicated immature, intermediate, and myeloid-primed groups cluster genes. **(C)** Slingshot trajectory analysis of unstimulated/stimulated MPP3 scRNA-seq dataset with identified branches. **(D)** Velocity and pseudotime analysis of unstimulated/stimulated MPP3 scRNA-seq dataset. 0 denotes pseudotime start, and 1 indicates pseudotime end.

**Figure S3.**
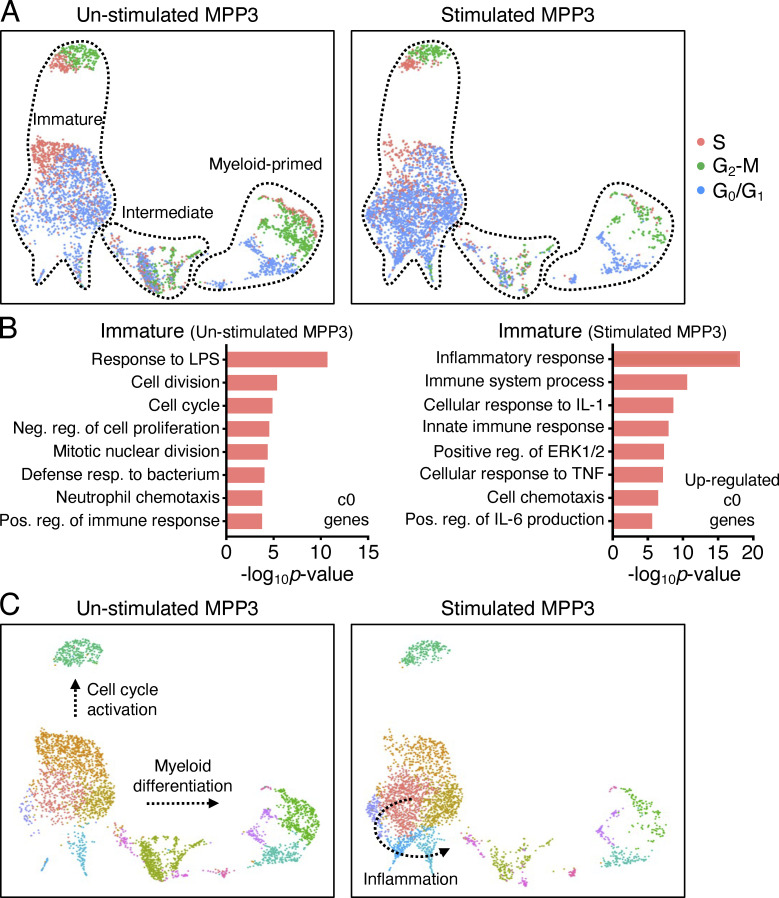
**MPP3 heterogeneity. (A)** Separated UMAP of unstimulated/stimulated MPP3 scRNA-seq dataset showing cell cycle distribution. The cell count per cluster is presented in Table S3. **(B)** GO analyses of immature cluster 0 genes in unstimulated/stimulated MPP3 scRNA-seq dataset. Only upregulated genes (>four-fold increase) are shown for stimulated MPP3. The full list of GO analyses of all clusters is presented in Table S3. **(C)** Separate UMAP of unstimulated/stimulated MPP3 scRNA-seq dataset with predicted Slingshot trajectories. Results are from isolated MPP3 cultured for 6 h ± LPS and Pam3CSK4 (L/P) stimulation.

**Figure 3.**
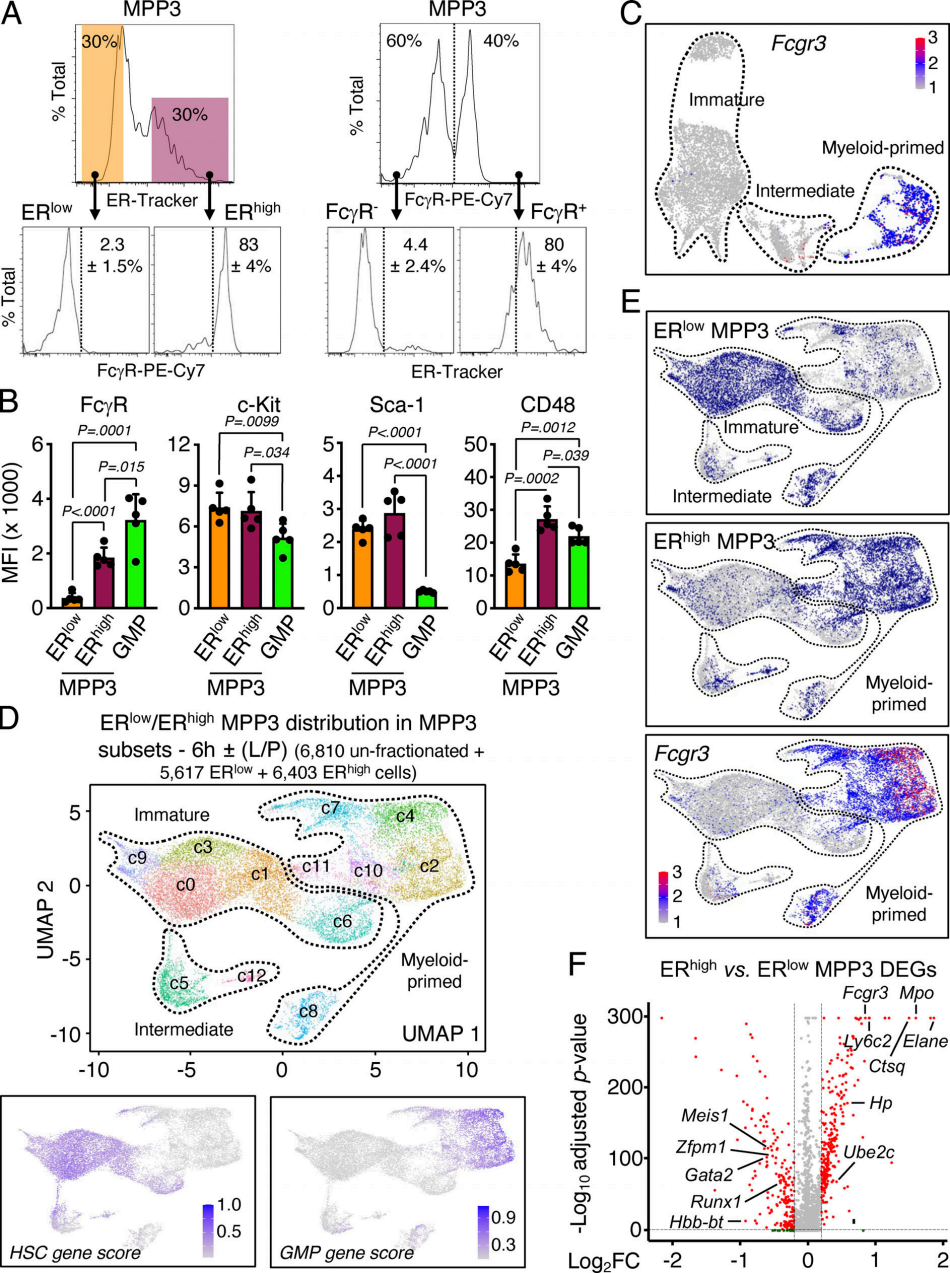
**Identification of distinct MPP3 subsets. (A)** Characterization of MPP3 subsets with representative FACS plots and quantification of FcγR^+^ frequency in ER^low^/ER^high^ MPP3 (left) and ER^high^ frequency in FcγR^−^/FcγR^+^ MPP3 (right) in three independent experiments. **(B)** Quantification of surface marker expression in MPP3 subsets and GMPs. Results are shown as mean fluorescence intensity (MFI). Data are means ± SD (three independent experiments), and significance was assessed by a two-tailed unpaired Student’s *t* test. **(C)** UMAP of un-stimulated/stimulated MPP3 scRNA-seq dataset showing *Fcgr3* expression. **(D)** UMAP of harmonized unstimulated/stimulated ER^low^ MPP3, ER^high^ MPP3, and total MPP3 scRNA-seq datasets with experimental scheme (left) and gene module scoring for HSC and GMP genes (bottom). Results incorporate isolated ER^low^ and ER^high^ MPP3 cultured for 6 h ± LPS and Pam3CSK4 (L/P) stimulation. **(E)** Projection of ER^low^ MPP3 (top), ER^high^ MPP3 (middle), and *Fcgr3* expression (bottom) on the UMAP of harmonized unstimulated/stimulated ER^low^/ER^high^/total MPP3 scRNA-seq datasets. **(F)** Volcano plot of DEGs between ER^high^ MPP3 vs. ER^low^ MPP3 scRNA-seq datasets showing representative examples. The full list of DEGs is presented in Table S4 A.

To gain a better understanding of MPP3 heterogeneity and response to inflammatory stimulation, we performed droplet-based scRNA-seq (10X Genomics) analyses on MPP3 stimulated ±LPS/Pam3CSK4 for 6 h. Data from unstimulated and stimulated MPP3 were harmonized by nearest neighbor integration and 13 different clusters were identified in uniform manifold approximation and projection (UMAP) representation (Fig. 2 A). To unravel the molecular structure of the MPP3 compartment and its organization along a continuum of differentiation, we used HSC and GMP conserved gene signature lists that we extracted from three independent scRNA-seq analyses of LK (Lin^−^/c-Kit^+^) and LSK (Lin^−^/Sca-1^+^/c-Kit^+^) datasets (Table S2). This approach allowed us to categorize the 13 MPP3 clusters into three distinct groups: an HSC gene-enriched immature group, a GMP gene-enriched myeloid-primed group, and a metabolically activated intermediate group (Fig. 2 A). Gene ontology (GO) and Slingshot analyses provided in-depth annotation of the clusters composing each group. Within the immature group, cluster 5 exhibited molecular features of cycling cells, which was directly supported by cell cycle distribution analyses and aligned along a predicted “cell cycle activation” path progressing through cluster 1, while cluster 9, cluster 8, and cluster 7 all displayed strong features of inflammatory response and were aligned along a predicted “inflammation” path (Fig. 2, B and C; and Fig. S3, A and B). Interestingly, cluster 3 in the intermediate group had clear features of metabolic activation, while cluster 12, cluster 10, cluster 4, and cluster 6 within the myeloid-primed group all showed strong signatures of mature myeloid cells and were aligned along a predicted “myeloid differentiation” path progressing through cluster 2 (Fig. 2, B and C; and Table S3). RNA velocity-based pseudotime analyses supported the myeloid differentiation trajectory from the immature group to the myeloid-primed group, with cell cycle distribution analyses confirming the progressive activation status of these clusters (Fig. 2 D and Fig. S3 A). Short-term 6-h inflammatory stimulation particularly amplified the “inflammation” branch, with upregulation of inflammatory response genes already detectable in stimulated cluster 0 (Fig. 3, B and C; and Table S3). Altogether, these results resolve the molecular heterogeneity of the MPP3 compartment with constitutive cell cycle activation and myeloid differentiation trajectories, and inducible production of inflammatory subsets upon stimulation.

### MPP3 are functionally heterogeneous

**Figure 4.**
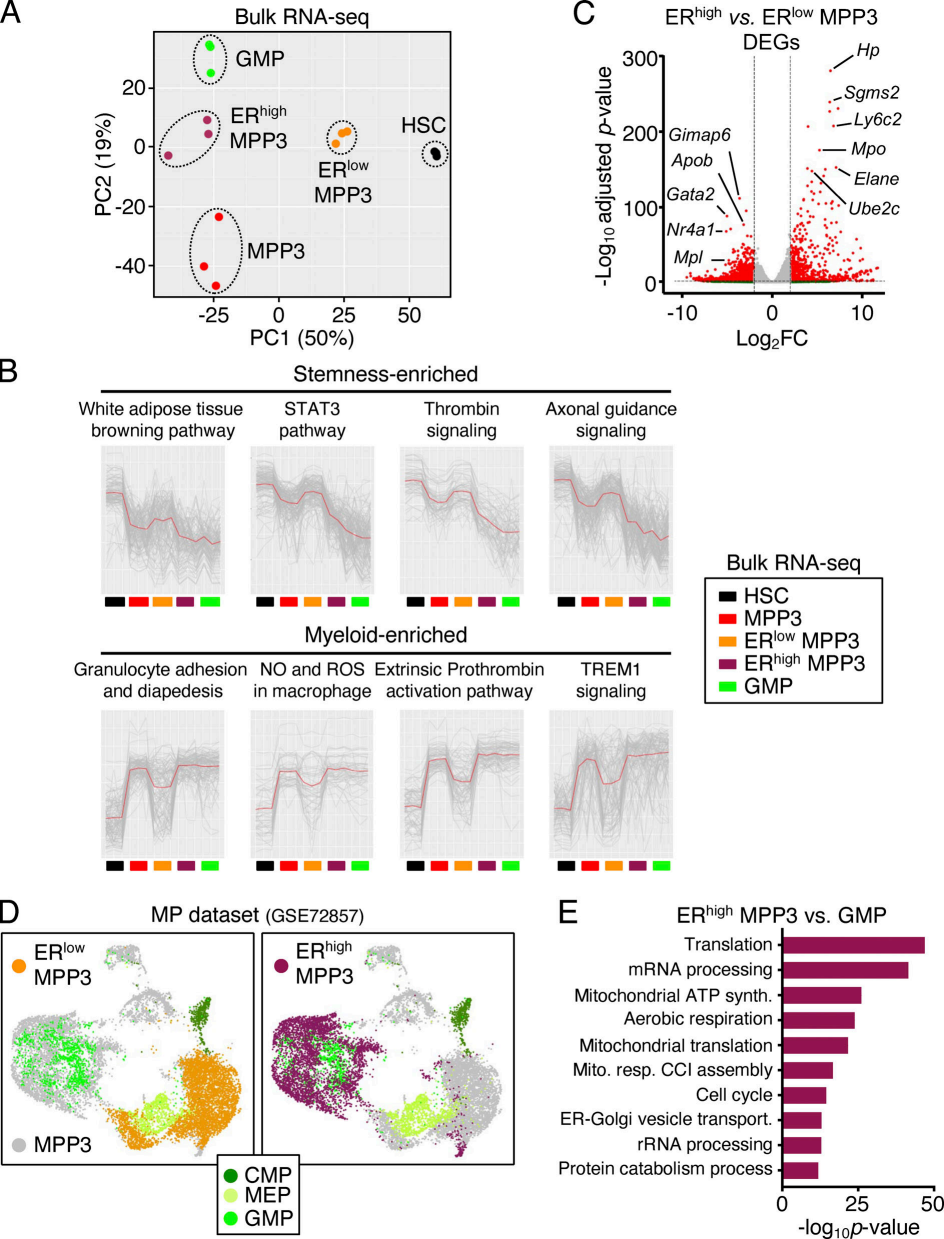
**Molecular characterization of MPP3 subsets. (A)** Principal component (PC) analysis of HSC, MPP3, ER^low^ MPP3, ER^high^ MPP3, and GMP bulk RNA-seq dataset. **(B)** K-means clustering analysis of highly variable genes in HSC, MPP3, ER^low^ MPP3, ER^high^ MPP3, and GMP bulk RNA-seq dataset showing representative enriched pathways. **(C)** Volcano plot of DEGs between ER^high^ MPP3 vs. ER^low^ MPP3 bulk RNA-seq dataset showing representative examples. The full list of DEGs is presented in Table S4 B. **(D)** UMAP of harmonized scRNA-seq datasets projecting unstimulated/stimulated MPP3 and ER^high^/ER^low^ MPP3 subsets onto a published myeloid progenitor (MP) dataset (GSE72857); MEP, megakaryocyte/erythrocyte progenitor. **(E)** GO analyses of ER^high^ MPP3 vs. GMP scRNA-seq datasets. The full list of DEGs is presented in Table S4 C.

We next investigated the function and regulation of the newly identified secretory ER^high^ MPP3 subset. Strikingly, ER^high^ MPP3 were almost exclusively FcγR^+^ like GMPs, but still differed from GMPs in their expression of all the other HSPC markers (Fig. 3, A and B). Conversely, the FcγR^+^ fraction of MPP3, which corresponds to myeloid-primed clusters in scRNA-seq analyses, was almost entirely ER^high^, while the FcγR^−^ fraction of MPP3, which corresponds to immature/intermediate clusters in scRNA-seq analyses, was almost entirely ER^low^ (Fig. 3, A and C). To further characterize ER^high^ and ER^low^ MPP3, we performed scRNA-seq analyses on those isolated subsets ± LPS/Pam3CSK4 stimulation, which we integrated with our previous unfractionated MPP3 scRNA-seq dataset (Fig. 3 D). These analyses directly showed that ER^high^ MPP3 corresponded to the *Fcgr3*-expressing myeloid-primed group and ER^low^ MPP3 to the HSC-like immature group (Fig. 3 E). Consistently, differentially expressed gene (DEG) analyses showed preferential expression of mature neutrophil genes like *Mpo* and *Elane* in ER^high^ MPP3, and immature HSC genes like *Gata2* and *Meis1* in ER^low^ MPP3 (Fig. 3 F and Table S4 A). Bulk RNA-seq and principal component analyses confirmed the similarity of ER^low^ MPP3 with HSCs and ER^high^ MPP3 with GMPs, with unfractionated MPP3 having a mixed gene identity (Fig. 4 A). K-means clustering analyses conducted on the bulk RNA-seq dataset using sets of highly variable genes (HVGs; P value of random permutation test <0.05) showed a drastic change of expression across these different cell types and further reinforced the clustering of ER^high^ MPP3 with GMPs and ER^low^ MPP3 with HSCs (Fig. 4 B). DEG analyses also showed a similar pattern to scRNA-seq analyses with mature neutrophil genes preferentially enriched in ER^high^ MPP3 and immature HSC genes preferentially enriched in ER^low^ MPP3 (Fig. 4 C and Table S4 B). Finally, integration of our scRNA-seq data with a previously published myeloid progenitor dataset (Paul et al., 2015) showed co-clustering of ER^high^ MPP3 with GMPs in UMAP representation, with ER^low^ MPP3 located in a separate area linked to GMP, common myeloid progenitors (CMP), and megakaryocyte/erythroid progenitors (MEP; Fig. 4 D). However, ER^high^ MPP3 still differed from GMPs in their molecular profile, with GO analyses indicating preferential expression of mRNA processing, mitochondrial energetics, and ER–Golgi vesicle transport genes in the secretory MPP3 subset (Fig. 4 E and Table S4 C). These molecular results indicate that ER^high^ MPP3 have the identity of a differentiation intermediary heralding GMP commitment, while ER^low^ MPP3 display broad myeloid differentiation potential, characteristic of an immature MPP.

**Figure 5.**
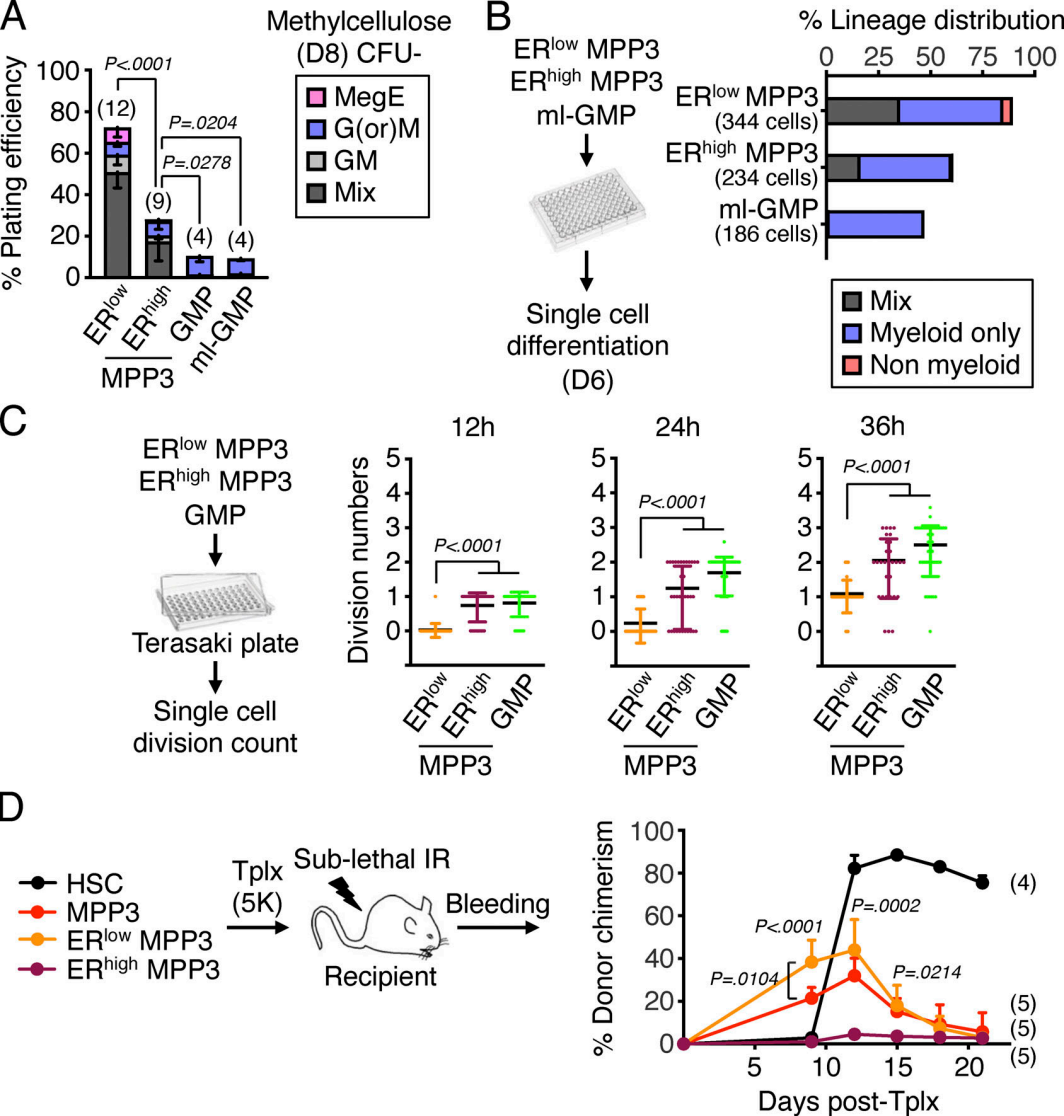
**Functional characterization of MPP3 subsets. (A)** Myeloid differentiation of ER^low^ MPP3, ER^high^ MPP3, GMP, and ml-GMP in methylcellulose assays. Results were scored after 8 d (D8) in three independent experiments. Mix, mixture of all lineages; GM, granulocyte/macrophage; G(or)M, granulocyte or macrophage; MegE, megakaryocyte/erythrocyte. **(B)** Single cell in vitro lineage differentiation assay of ER^high^/ER^low^ MPP3 subsets and ml-GMP with flow cytometry identification after 6 d (D6) in culture. A total of 384 single cells were assessed in four independent experiments with data expressed as a percentage of mix, myeloid only, and non-myeloid lineage output. Myeloid only, CD45^+^/Mac-1^+^/Gr-1^+^ myeloid cells; Non-myeloid, combination of CD45^+^/Mac-1^−^/Gr-1^−^/CD41^+^/CD61^+^ and manually counted megakaryocytes, CD45^+^/Mac-1^−^/Gr-1^−^/CD41^−^/CD61^+^/CD71^+^ erythroid cells and CD45^+^/Mac-1^−^/Gr-1^−^/CD41^−^/CD71^−^/FcεRI^+^ mast cells; Mix, both myeloid and non-myeloid output. **(C)** Single-cell in vitro division assay of ER^high^/ER^low^ MPP3 subsets and GMPs in Terasaki plates with an assessment of cell division after 12–36 h in culture. A total of 160 single cells were assessed in three independent experiments with data expressed as a scatter dot plot (bar, mean). **(D)** Short-term in vivo lineage tracing assay with the experimental scheme for the transplantation (tplx) of 5,000 cells into each sub-lethally irradiated (IR) recipient, and quantification of donor chimerism in PB over time. Significance was calculated between mice transplanted with ER^low^ or ER^high^ MPP3 unless otherwise indicated. Data are means ± SD, and significance was assessed by a two-tailed unpaired Student’s *t* test.

**Figure 6.**
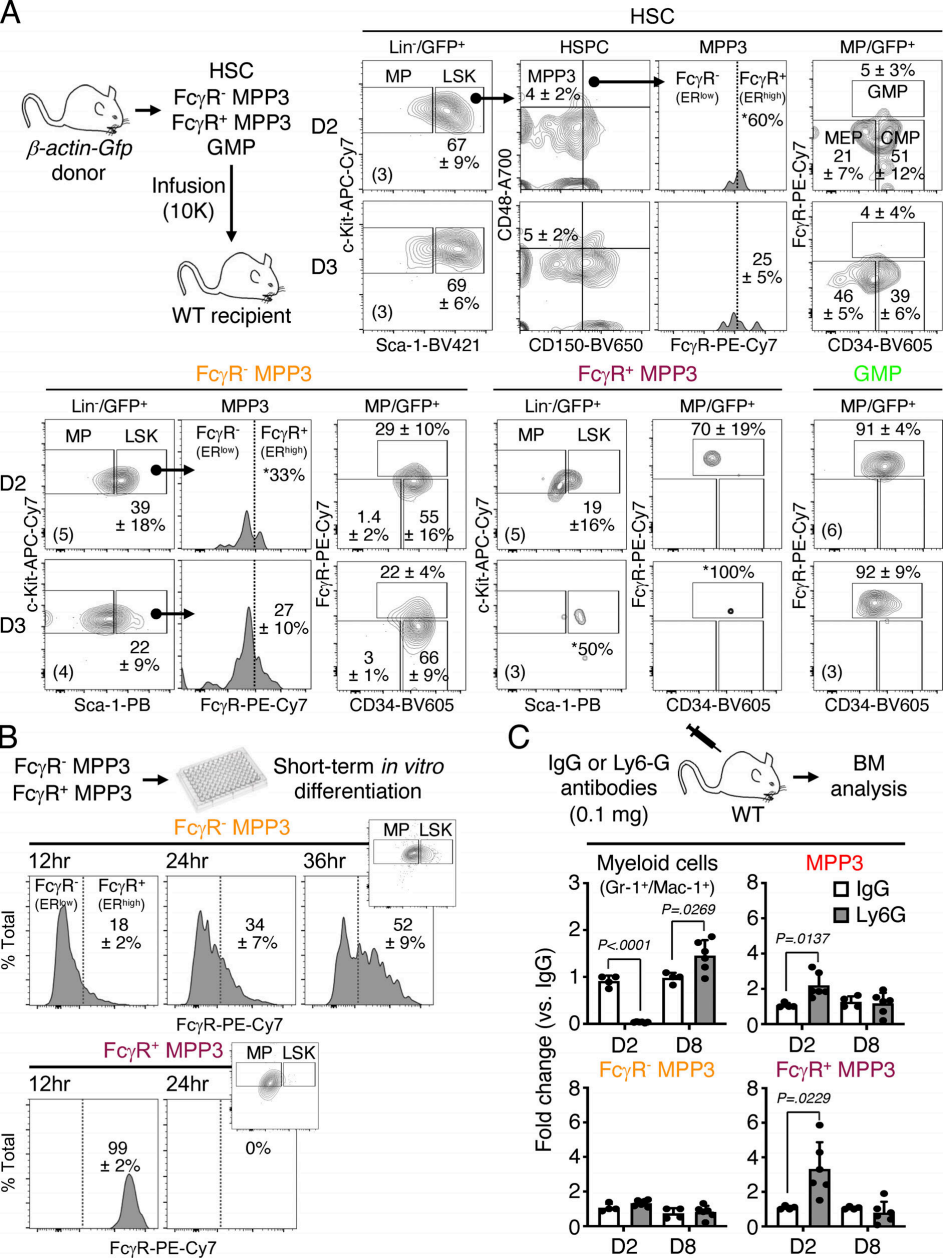
**Differentiation potential of MPP3 subsets. (A)** Short-term in vivo differentiation assays. Donor cells were isolated from *β-actin-Gfp* mice and infused into WT recipients (10,000 cells per mouse). GFP^+^ donor-derived cells were analyzed for LSK (Lin^−^/Sca-1^+^/c-Kit^+^) and MP (Lin^−^/Sca-1^−^/c-Kit^+^) contributions at 2 and 3 d (D) after infusion. Representative FACS plots and quantification of donor-derived frequencies are shown, with the numbers of infused recipients per population and timepoint indicated in parentheses. *, contribution detected only in one of the infused recipients. **(B)** Short-term in vitro differentiation of FcγR^−^ and FcγR^+^ MPP3 subsets. Cells (2,000 per well, three independent experiments) were cultured for 12, 24, and 36 h and analyzed for HSPC markers. Representative FACS plots and quantification of FcγR^high^ frequencies are shown; small insert show LSK/MP distribution at the same times. **(C)** Expansion of FcγR^−^ and FcγR^+^ MPP3 subsets during myeloid regeneration. **(D)** WT mice were injected with control IgG or anti-Ly6G depleting antibodies and analyzed for changes in the indicated BM populations after 2 and 8 d (D). Results are expressed as fold changes in population size at each time point compared to IgG-treated mice (two independent experiments).

To directly probe the differentiation potential of MPP3 subsets, we next performed a series of in vitro and in vivo functional investigations. Compared with ER^low^ MPP3, ER^high^ MPP3 had significantly lower colony-forming capacity in both methylcellulose and single cell differentiation assays in liquid culture and were more committed to myeloid differentiation (Fig. 5, A and B). ER^high^ MPP3 also had faster division kinetics than ER^low^ MPP3 in Terasaki single-cell assays, akin to GMPs (Fig. 5 C), but still appeared distinct from GMPs, even from the most potent (Ly6C^−^/CD115^−^) multilineage GMP (ml-GMP) subset (Fig. S1 A; Olsson et al., 2016), as they retained some residual ability to give rise to mixed colonies in vitro (Fig. 5, A and B). However, in vivo, transplantation into sublethally irradiated recipients showed that ER^high^ MPP3 were almost entirely devoid of reconstitution potential compared with ER^low^ MPP3 (Fig. 5 D). To extend the characterization of MPP3 subsets, we next took advantage of the association between FcγR expression and ER volume to track the differentiation path of FcγR^−^ and FcγR^+^ MPP3 isolated from *β-actin-Gfp* mice upon infusion into non-irradiated recipient mice (Fig. 6 A). Strikingly, 2 or 3 d after infusion, FcγR^−^ MPP3 gave rise to both FcγR^−^ and FcγR^+^ MPP3 and all myeloid progenitors, while FcγR^+^ MPP3 did not persist or expand, which further confirmed their lack of engraftment potential. In contrast, in the same assay, GMPs maintained themselves, while HSCs did not yet appear capable of producing MPP3 or GMP output (Fig. 6 A). In short-term liquid culture differentiation assays, FcγR^−^ MPP3 similarly produced both FcγR^−^ and FcγR^+^ MPP3 as well as a strong myeloid progenitor compartment, while FcγR^+^ MPP3 were unable to produce FcγR^−^ MPP3 and quickly differentiated into myeloid progenitors (Fig. 6 B). These results are consistent with a model whereby myeloid-primed and secretory FcγR^+^/ER^high^ MPP3 are a short-lived, proliferating, and non-engrafting transitional population preceding GMP commitment, while immature FcγR^−^/ER^low^ MPP3 represent the true multipotent part of the MPP3 compartment capable of generating FcγR^+^/ER^high^ MPP3 and differentiating toward other myeloid lineage fates.

### Secretory MPP3 stimulate myelopoiesis

**Figure 7.**
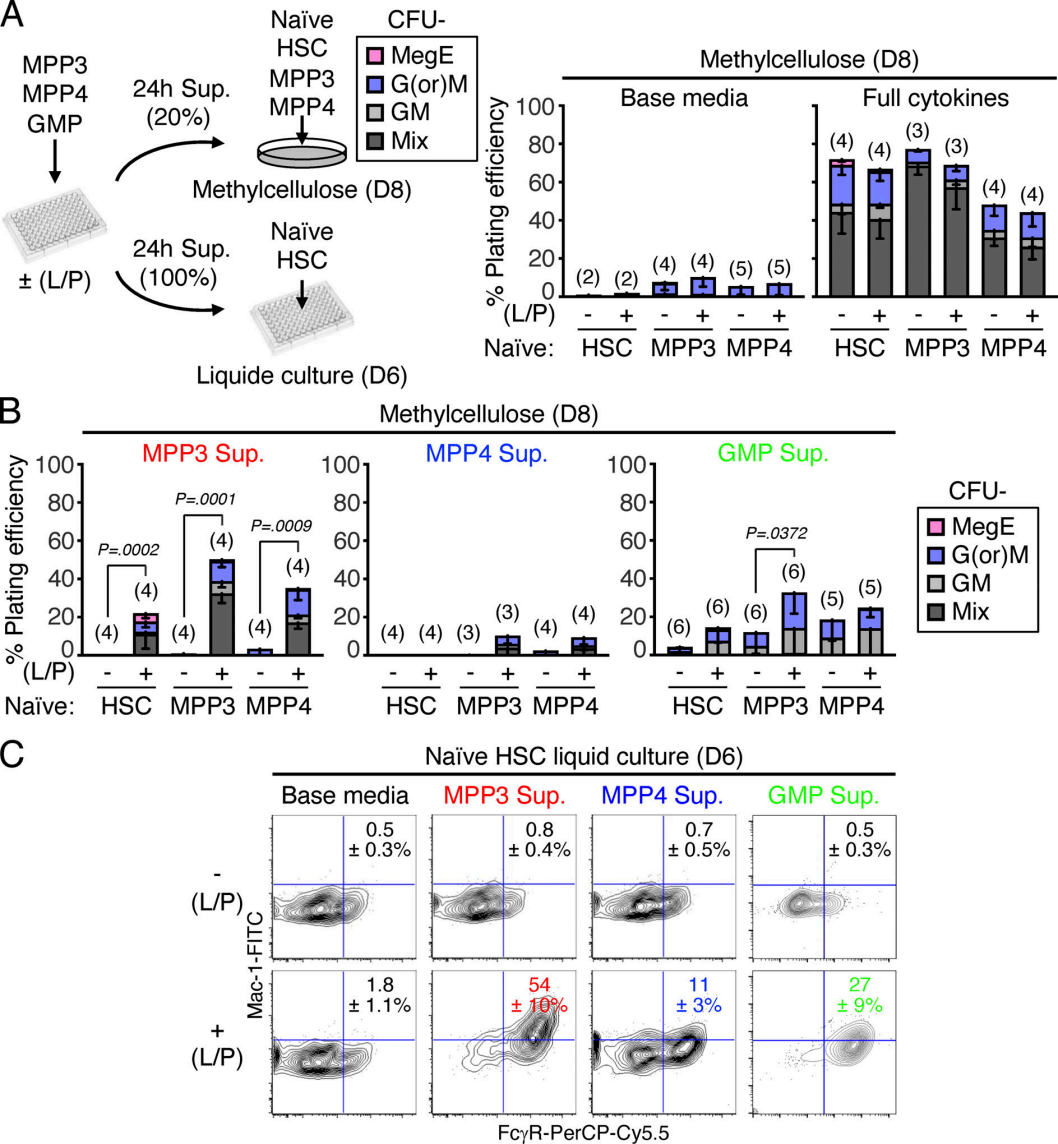
**Pro-myeloid differentiation effect of MPP3 secretion. (A)** Experimental scheme to assess the pro-myeloid differentiation effect of MPP3, MPP4, and GMP supernatants on naïve HSCs, MPP3 and MPP4 plated either in methylcellulose with 20% supernatant or liquid cultures in 100% supernatant. Supernatants were collected upon culture of 10,000 MPP3, MPP4, or GMP for 24 h in 150 µl base media ± LPS/Pam3CSK4 (L/P) stimulation. CFU in methylcellulose assays were scored after 8 d (D8) and differentiating cells in liquid cultures were analyzed by flow cytometry after 6 d (D6). Results from control methylcellulose assays performed with only base media or base media with full cytokine cocktail are shown on the right. **(B)** Effect of MPP3, MPP4, and GMP supernatants (Sup.) on naïve HSCs, MPP3, and MPP4 differentiation in methylcellulose. Results from colonies scored at D8 are shown; Mix, mixture of all lineages; GM, granulocyte/macrophage; G(or)M, granulocyte or macrophage; MegE, megakaryocyte/erythrocyte. **(C)** Effect of MPP3, MPP4 and GMP supernatants on naïve HSCs in liquid cultures. Representative FACS plots and quantification of Mac-1^+^/FcγR^+^ myeloid cell frequencies after 6 d in three independent experiments are shown. Un-stim., un-stimulated; Stim., stimulated. Data are means ± SD, and significance was assessed by a two-tailed unpaired Student’s *t* test.

**Figure 8.**
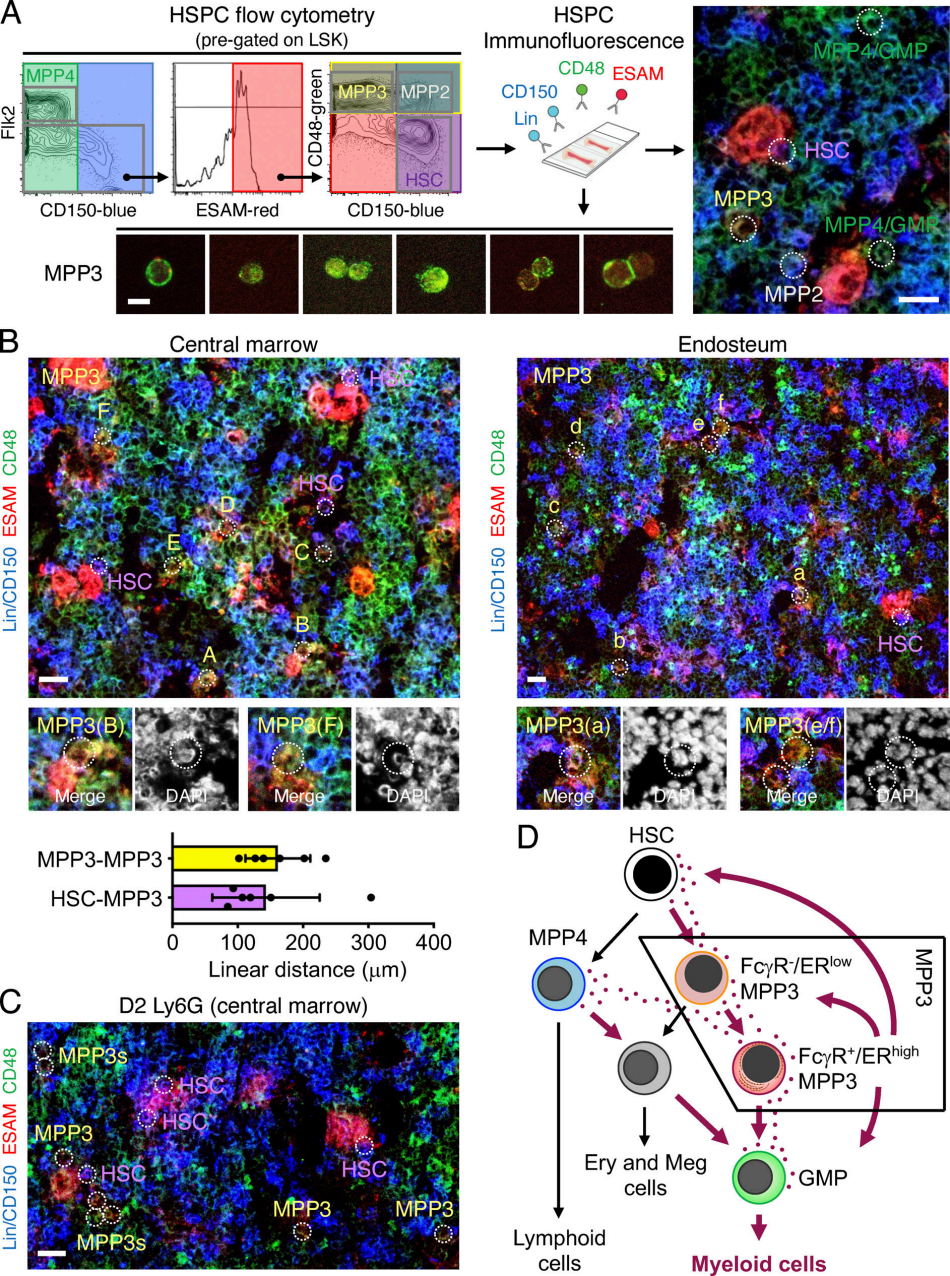
**MPP3 are localized in the vicinity of other HSPCs in the BM cavity. (A)** Immunofluorescence staining strategy to identify HSPC populations in situ on bone sections with flow cytometry showing the simplified three-color staining scheme used (left), representative examples of stained MPP3 isolated by flow cytometry (bottom; scale bar, 10 µm), and a representative example of stained BM section (right; scale bar, 20 µm). Populations are indicated by white dotted line circles, and while HSC (purple), MPP2 (white), MPP3 (yellow) can be distinguished from the rest of the BM (blue) and megakaryocytes/megakaryocytic lineage (red) cells. MPP4 (green) are largely overlapping with GMPs in this staining scheme. Also, note the relatively spotty CD48 surface expression on MPP3. **(B)** Representative images of in situ immunofluorescence staining of HSPCs in the central marrow cavity (left) and at the endosteum (right), and quantification of linear distance between the indicated populations (bottom, 30 MPP3/MPP3 and 34 HSC/MPP3 pairs were counted in six independent experiments). MPP3 (yellow) are indicated by white dotted line circles, with magnified images of the indicated cells shown below with DAPI counterstain. HSCs (purple) are also denoted at both locations. Scale bar, 20 μm. **(C)** Representative image of in situ immunofluorescence staining of HSPCs in the central marrow cavity of anti-Ly6G antibody-treated mice at 2 d (D2) after injection showing the expansion and closest proximity of MPP3 with HSCs (white dotted line circles) in regenerative conditions. Scale bar, 20 μm. **(D)** Model depicting myeloid differentiation trajectories in early hematopoietic hierarchy and the role of secretory FcγR^+^/ER^high^ MPP3 subset in amplifying HSPC myeloid commitment through autocrine/paracrine regulation in the BM microenvironment. Ery, erythrocytes; Meg, megakaryocytes.

We next directly tested whether secretory FcγR^+^/ER^high^ MPP3 had a role in rapidly amplifying myeloid cell production upon demand. We first used a well-established model of in vivo myeloid regeneration driven by granulocyte depletion upon anti-Ly6G antibody injection, in which we previously demonstrated transient MPP3 expansion prior to GMP and myeloid cell expansion (Kang et al., 2020). Strikingly, we found that the expansion of regenerative MPP3 exclusively resulted from increased FcγR^+^ MPP3 (Fig. 6 C), which confirms in vivo the role of this secretory subset as an amplification compartment for myelopoiesis. We next used supernatants from MPP3, MPP4, and GMP cultured for 24 h in vitro ± LPS/Pam3CSK4 stimulation to perform differentiation assays with naïve HSPCs in methylcellulose and liquid culture (Fig. 7 A). For practical reasons, we used supernatants from unfractionated MPP3 as a surrogate for ER^high^ MPP3 and unfractionated MPP3 as naïve cells to readout the full differentiation potential of this compartment, including the multipotent FcγR^−^/ER^low^ MPP3 subset. Remarkably, supernatants from stimulated MPP3 massively induced myeloid colony formation in methylcellulose in the range of full cytokine stimulation, with the production of GM/MegE mixed colonies not only from naïve HSCs but also from naïve MPP3 and MPP4 (Fig. 7 B). In contrast, supernatants from stimulated MPP4 barely elicited myeloid differentiation from any of the naïve populations, while supernatants from stimulated GMP modestly expanded GM-committed colonies and to a much lower extent than stimulated MPP3 supernatants. Liquid culture experiments confirmed these results with supernatants from stimulated MPP3, but not MPP4, inducing myeloid differentiation with robust production of Mac-1^+^/FcγR^+^ myeloid cells from naïve HSCs, and supernatants from stimulated GMPs also enhancing myeloid cell production but to a much lesser extent than supernatants from stimulated MPP3 (Fig. 7 C). To provide further support for the local paracrine/autocrine effect of MPP3 secretion, we finally developed a novel immunofluorescence imaging panel on thin 7-µm sections of BM to visualize MPP3 in their native microenvironment (Fig. 8 A). Using a combination of blue Lin/CD150, red ESAM, and green CD48 antibodies, we were able to distinguish HSC (purple), MPP2 (white), MPP3 (yellow), and MPP4/GMP (green) cells from the rest of the BM (blue) and megakaryocytes/megakaryocytic lineage (red) cells. However, with this staining limited to three fluorophores and DAPI, we could neither distinguish MPP4 from GMPs nor the different types of MPP3 subsets. Importantly, MPP3 were consistently found in the vicinity of other HSPCs both at the endosteum and in the central marrow cavity, with an average of 143 ± 82 µm linear distance between the nearest HSC and MPP3 and 162 ± 50 µm between the nearest MPP3 (Fig. 8 B). These results are consistent with the long-range migration of MPP away from HSCs recently reported with another imaging approach (Wu et al., 2022) and support the idea that MPP3 secretion could act locally on nearby HSPCs. In fact, in situ BM imaging of day 2 post-Ly6G depletion bones showed increased numbers of MPP3 in close proximity to HSCs in this regenerative condition, further supporting the function of MPP3 secretion in engaging emergency myelopoiesis (Fig. 8 C). Collectively, these findings identify a novel self-reinforcing regulatory function for a secretory FcγR^+^/ER^high^ MPP3 subset (Fig. 8 D), which controls myelopoiesis through intrinsic lineage-priming toward GMP differentiation and cytokine production in the BM niche, thereby likely amplifying myeloid cell production from other HSPC populations via autocrine and paracrine effects.

### Secretory MPP3 are specifically expanded in myeloid leukemia

**Figure 9.**
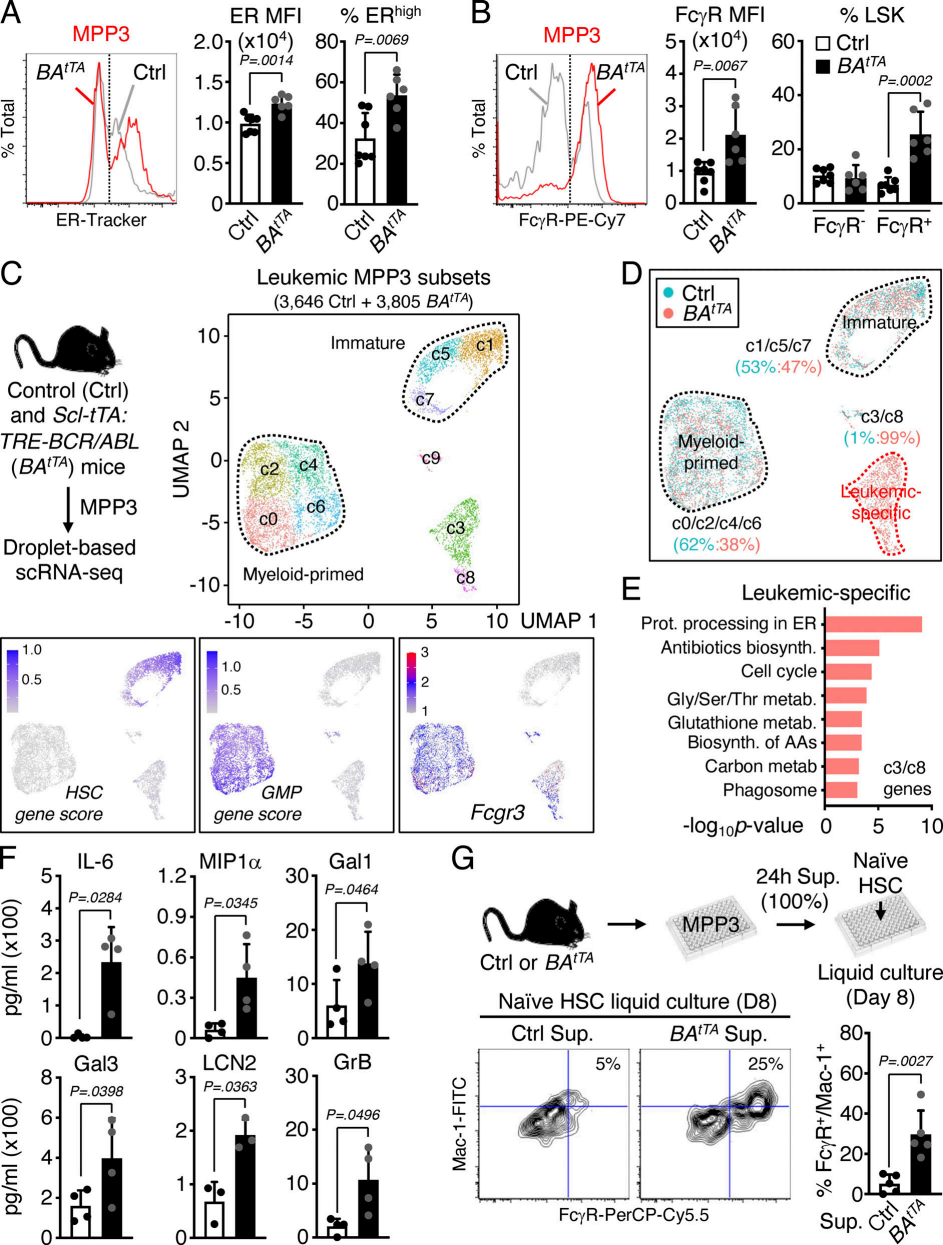
**Constant secretion and myeloid amplification from leukemic FcγR**^**+**^**/ER**^**high**^ **MPP3. (A and B)** Quantification of (A) ER content (three independent experiments) and (B) FcγR surface expression (four independent experiments) in Ctrl and *BA*^*tTA*^ MPP3; MFI, mean fluorescence intensity. **(C)** UMAP representation of Ctrl and *BA*^*tTA*^ MPP3 scRNA-seq dataset with experimental scheme (left), gene module scoring for HSC and GMP genes (bottom left and center), and *Fcgr3* expression (bottom right). **(D)** UMAP representation of Ctrl/*BA*^*tTA*^ MPP3 scRNA-seq dataset showing color coded ratio of Ctrl:*BA*^*tTA*^ cells in major immature, myeloid-primed, and leukemic-specific groups. **(E)** KEGG pathway analysis of leukemic-specific cluster genes; Prot., protein; biosynth., biosynthesis; metab., metabolism; Gly, glycine; Ser, serine; Thr, threonine; AAs; amino acids. **(F)** Quantification of IL-6, MIP1α, Gal1, Gal3, LCN2, and GrB levels in unstimulated Ctrl and *BA*^*tTA*^ MPP3 supernatants (four independent experiments). **(G)** Pro-myeloid differentiation effect of *BA*^*tTA*^ MPP3 supernatant (Sup.) on naïve HSCs analyzed after 8 d (D) of liquid culture. Experimental scheme, representative FACS plots, and quantification of Mac-1^+^/FcγR^+^ frequencies are shown (four independent experiments). Supernatants were collected upon culture of 10,000 Ctrl or *BA*^*tTA*^ MPP3 for 24 h in 150 µl base media. Data are means ± SD, and significance was assessed by a two-tailed unpaired Student’s *t* test.

**Figure S4.**
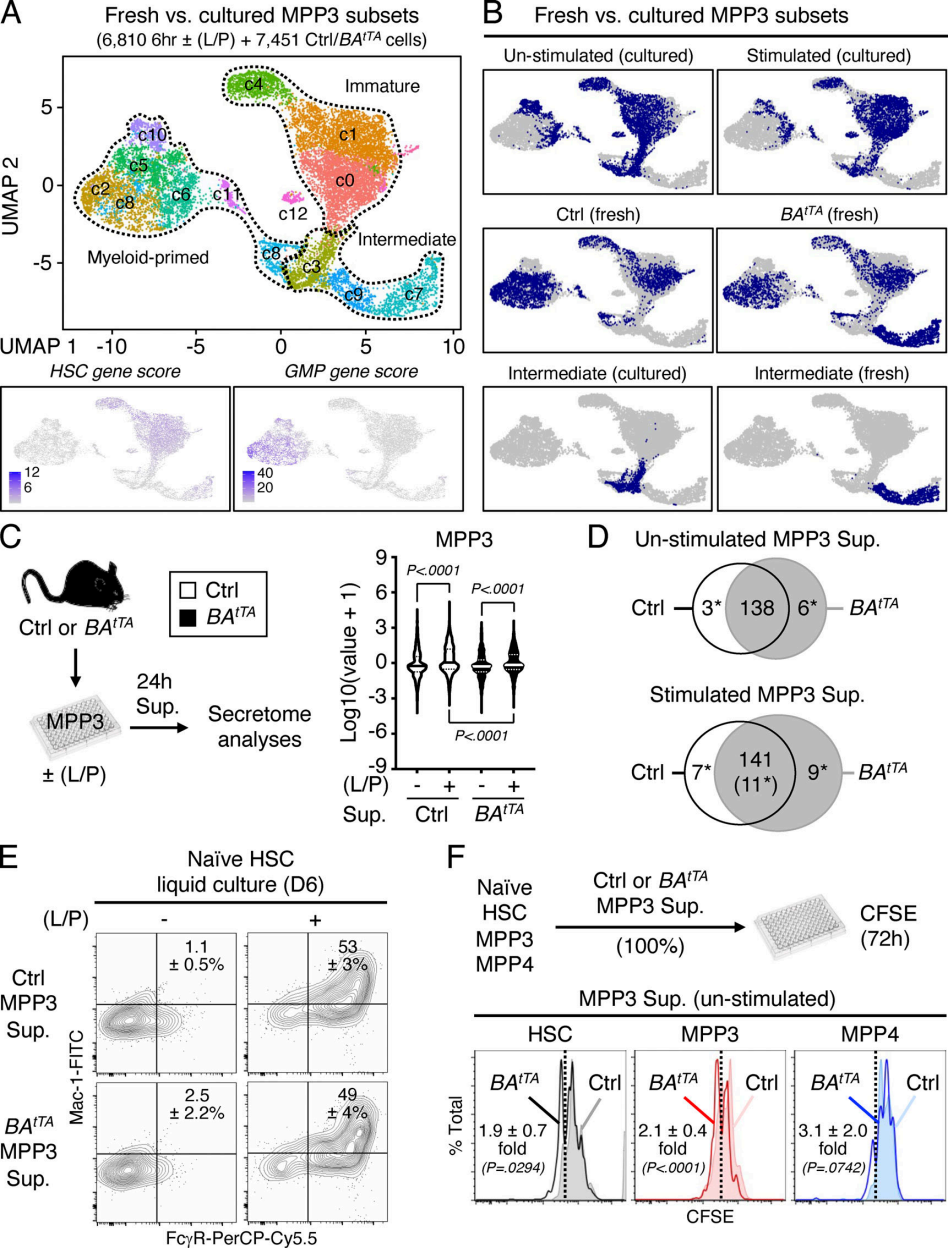
**Molecular rewiring and constitutive cytokine secretion of leukemic MPP3. (A and B)** Comparison of freshly isolated and cultured MPP3 with (A) UMAP of harmonized unstimulated/stimulated MPP3 and freshly isolated Ctrl/*BA*^*tTA*^ MPP3 scRNA-seq datasets with gene module scoring for HSC and GMP genes (bottom) and (B) single projection of each dataset and specific metabolic intermediate clusters. **(C and D)** Secretome analyses of unstimulated/stimulated Ctrl and *BA*^*tTA*^ MPP3 supernatants (Sup.) with (C) experimental scheme and violin plots of secreted cytokine intensity (four independent experiments; bar, median; dotted line, quartiles), and (D) Venn diagrams showing similarly and differentially secreted cytokines (*, significant change). Supernatants were collected upon the culture of 10,000 Ctrl or *BA*^*tTA*^ MPP3 for 24 h in 150 µl base media ± LPS/Pam3CSK4 (L/P) stimulation. The full list of differentially secreted cytokines is provided in Table S5. **(E)** Differentiation of naïve HSCs in Ctrl and *BA*^*tTA*^ MPP3 supernatants analyzed after 6 d (D) of liquid culture for myeloid cell markers. Representative FACS plots and quantification of Mac-1^+^/FcγR^+^ frequencies are shown (three independent experiments). **(F)** Effect of unstimulated Ctrl and *BA*^*tTA*^ MPP3 supernatants on naïve HSCs, MPP3, and MPP4 proliferation analyzed by CFSE dilution assay after 72 h in culture. Experimental scheme and representative FACS plots are shown. Dotted lines identify CSFE^low^ fast proliferative cells, and results indicate the proproliferative effect of *BA*^*tTA*^ MPP3 supernatant shown as fold change compared with Ctrl MPP3 supernatant (four independent experiments for HSC and MPP4, five independent experiments for MPP3). Data are means ± SD, and significance was assessed by a two-tailed unpaired Student’s *t* test.

Finally, we investigated the role of this newly identified myeloid amplification mechanism in malignant myelopoiesis. We used our inducible *Scl-tTA:TRE-BCR/ABL* (*BA*^*tTA*^) mouse model of myeloproliferative neoplasm, which we previously characterized for HSPC remodeling and MPP3 expansion associated with leukemic GMP cluster formation and myeloid cell production (Reynaud et al., 2011; Hérault et al., 2017; Kang et al., 2020). Strikingly, we found that leukemic MPP3 expansion in *BA*^*tTA*^ mice was entirely driven by the increase of FcγR^+^/ER^high^ MPP3 (Fig. 9, A and B). To gain molecular insights, we performed scRNA-seq analyses on MPP3 isolated from 11- to 13-wk-old age-matched control (Ctrl) and diseased *BA*^*tTA*^ mice (Fig. 9 C). Integrated UMAP and gene module scoring analyses identified the expected immature-like and myeloid-primed groups but also uncovered a *Fcgr3*-expressing leukemic-specific group composed of two clusters (cluster 3, cluster 8) that were 99% of *BA*^*tTA*^ origin (Fig. 9, C and D). Interestingly, this leukemic-specific group exhibited hallmark features of increased metabolism and biosynthetic processes, which were distinct from metabolic activation of normal MPP3 in culture and upon inflammatory stimulation (Fig. 9 E and Fig. S4, A and B). These molecular data demonstrate the amplification of a unique subset of metabolically activated FcγR^+^/ER^high^ MPP3 in leukemic conditions, probably as a direct consequence of BCR/ABL activity (Konig et al., 2007; Zhao et al., 2010; Barger et al., 2013).

**Figure S5.**
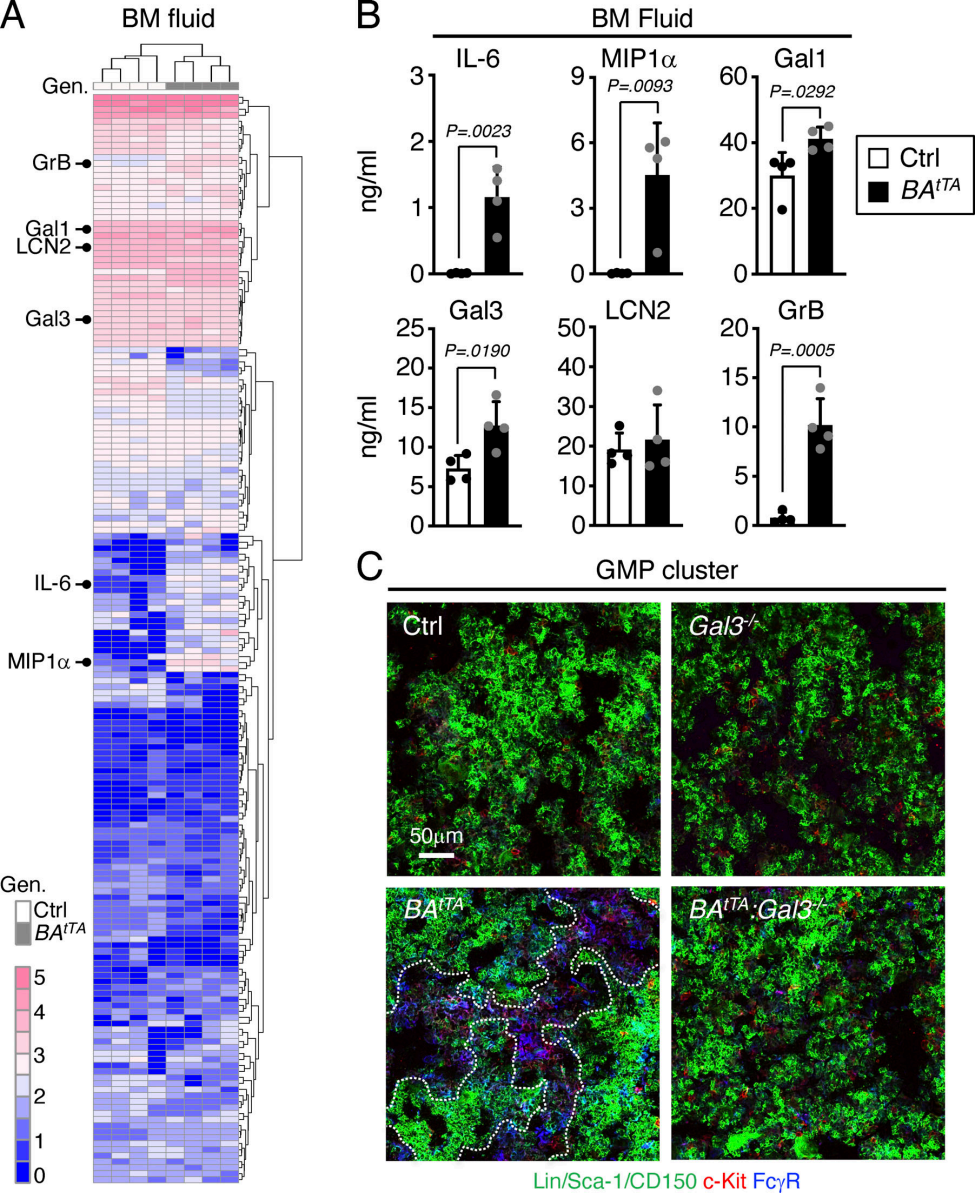
**Changes in the BM niche microenvironment. (A and B)** Analysis of the cytokines secreted in the BM fluid of Ctrl and *BA*^*tTA*^ mice with (A) heatmap of unsupervised clustering of BM fluid cytokine levels after quantile normalization (Gen; genotype), and (B) detailed quantification of IL-6, MIP1α, Gal1, Gal3, LCN2, and GrB levels. BM fluids were obtained by flushing four long bones (femur and tibia) of each mouse with 200 µl media and were analyzed with the Raybiotech 200 mouse cytokine array (four independent experiments). The six cytokines constitutively secreted by *BA*^*tTA*^ MPP3 are indicated on the left in A, and the full list of the cytokines differentially expressed in *BA*^*tTA*^ vs. Ctrl BM fluids is provided in Table S5. **(C)** Representative images of GMP immunofluorescence staining in Ctrl, *Gal3*^*−/−*^, *BA*^*tTA*^, *BA*^*tTA*^*:Gal3*^*−/−*^ BM. Dotted lines denote GMP clusters; Scale bar, 50 µm. Data are means ± SD, and significance was assessed by a two-tailed unpaired Student’s *t* test.

Next, we tested the secretory activity of leukemic MPP3 and collected supernatants from Ctrl and *BA*^*tTA*^ MPP3 cultured for 24 h in vitro ± LPS/Pam3CSK4 stimulation to perform secretome analyses and functional studies (Fig. S4 C). Both Ctrl and *BA*^*tTA*^ MPP3 secreted similar levels of cytokines upon stimulation with largely overlapping profiles and pro-myeloid differentiation effect of stimulated supernatants on naïve HSCs in liquid culture (Fig. S4, D and E). However, *BA*^*tTA*^ MPP3 constitutively secreted a unique set of six cytokines, some with well-known functions in myelopoiesis, composed of IL-6, MIP1α, Galectin 1 (Gal1), Galectin 3 (Gal3), Lipocalin 2 (LCN2), and Granzyme B (GrB; Fig. 9 F and Table S5; Mirantes et al., 2014). In fact, upon further culture (day 8), unstimulated *BA*^*tTA*^ MPP3 supernatants increased the proliferation of naïve HSC, MPP3, and MPP4, and enhanced myeloid differentiation from naïve HSCs in liquid culture, although to a lower extent and with slower kinetics than LPS/Pam3CSK4-stimulated supernatants at day 6 (Fig. 9 G and Fig. S4 F). Except for LCN2, the cytokines constitutively secreted by *BA*^*tTA*^ MPP3 were also found at elevated levels in the BM fluid of *BA*^*tTA*^ mice (Fig. S5, A and B; and Table S5). Collectively, these results demonstrate an amplification of secretory MPP3 in leukemic conditions, with constitutive secretion of known pro-myeloid differentiation cytokines that could play active roles in disease pathogenesis.

### Leukemic MPP3-secreted cytokines promote malignant myeloid cell production

**Figure 10.**
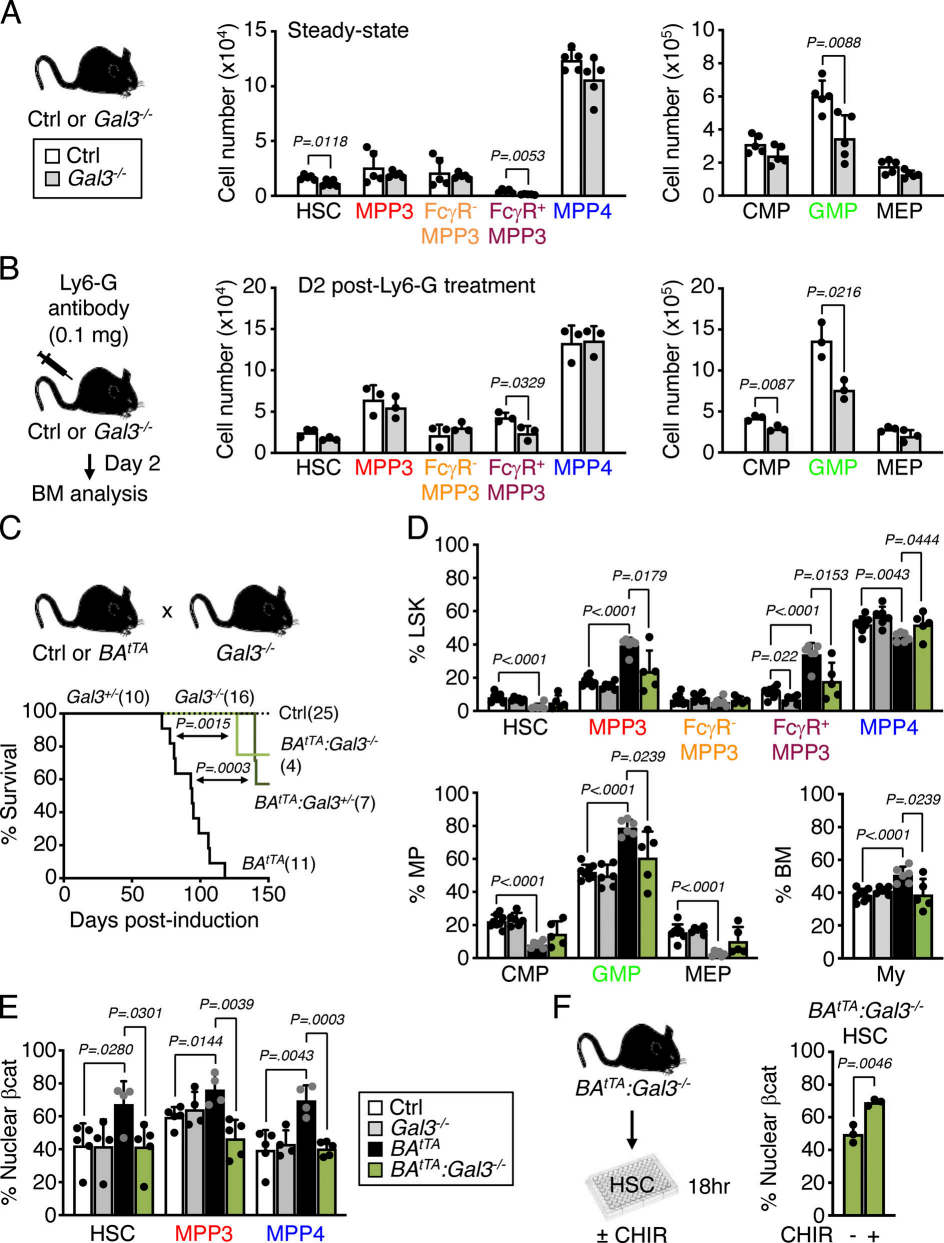
**Gal3 secretion contributes to myeloid amplification by leukemic MPP3. (A)** HSPC and myeloid progenitor population size in age-matched Ctrl *Gal3*^*+/+*^ and knockout *Gal3*^*−/−*^ mice (two independent experiments). **(B)** Changes in HSPC and myeloid progenitor population size in Ctrl and *Gal3*^*−/−*^ mice 2 d (D) after injection of anti-Ly6G depleting antibodies in one independent experiment. **(C)** Survival curve of *BA*^*tTA*^ mice with *Gal3* deletion. Results from Ctrl, *Gal3*^*+/−*^, *Gal3*^*−/−*^, *BA*^*tTA*^, *BA*^*tTA*^*:Gal3*^*+/−*^, and *BA*^*tTA*^*:Gal3*^*−/−*^ mice from five independent cohorts are shown; induction, doxycycline withdrawal. Significance was assessed by a Mantel-Cox test. **(D)** Changes in population size for HSPCs, myeloid progenitors, and mature myeloid cells (My, Mac-1^+^/Gr-1^+^) in 11- to 13-wk-old age-matched Ctrl, *Gal3*^*−/−*^, *BA*^*tTA*^, and *BA*^*tTA*^*:Gal3*^*−/−*^ mice. Results are expressed as a percentage of Lin^−^/Sca-1^+^/c-Kit^+^ (LSK), Lin^−^/Sca-1^−^/c-Kit^+^ (MP), and BM cells, and are from five independent cohorts. **(E)** Quantification of nuclear β-catenin (βcat) positive HSC, MPP3, and MPP4 in a subset of age-matched Ctrl, *Gal3*^*−/−*^, *BA*^*tTA*^, and *BA*^*tTA*^*:Gal3*^*−/−*^ mice shown in D. **(F)** Changes in the frequency of nuclearβcat-positive *BA*^*tTA*^*:Gal3*^*−/−*^ HSCs upon 18 h of in vitro treatment with the GSK3β inhibitor CHIR 99021 (CHIR, 30 µM) (two independent experiments). Data are means ± SD, and significance was assessed by a two-tailed unpaired Student’s *t* test except when indicated.

Among the cytokines constitutively secreted by leukemic MPP3, Gal3 is highly expressed in various cancers and is a negative prognostic factor for acute myeloid leukemia patients (Cheng et al., 2013; Liu and Rabinovich, 2005; Yamamoto-Sugitani et al., 2011). To test the importance of Gal3 secretion in myeloid amplification induced by leukemic MPP3, we crossed a previously published *Gal3*^*−/−*^ mouse line (Hsu et al., 2000) with *BA*^*tTA*^ mice. *Gal3*^*−/−*^ mice already showed reduced commitment toward myelopoiesis at steady state, with decreased HSC, FcγR^+^ MPP3, and GMP compartments, which persisted upon anti-Ly6G depletion treatment, with impaired FcγR^+^ MPP3 and GMP expansion in regenerative conditions at day 2 (Fig. 10, A and B). Remarkably, both heterozygote and homozygote *Gal3* deletion significantly extended the survival of *BA*^*tTA*^ mice with the reversion of the leukemic expansion of FcγR^+^ MPP3 and GMP, reduced GMP cluster formation, and restoration of the defective production of MPP4 in *BA*^*tTA*^*:Gal3*^*−/−*^ mice (Fig. 10, C and D; and Fig. S5 C). These results are strikingly similar to the amelioration of disease development we previously observed in *BA*^*tTA*^ mice upon deletion of IL-6 (Reynaud et al., 2011), another cytokine constitutively secreted by leukemic MPP3. Gal3 is known to activate Wnt/β-catenin signaling by inhibiting GSK3β (Song et al., 2009; Song et al., 2020), and we previously identified high Wnt/β-catenin activity in HSPCs as one of the key mechanisms driving MPP3 expansion and increased myelopoiesis in regenerative and leukemic conditions (Kang et al., 2020). Accordingly, we found a significant reduction in nuclear β-catenin levels in *BA*^*tTA*^*:Gal3*^*−/−*^ HSCs, likely owing to increased GSK3β activity caused by the loss of Gal3-mediated regulation (Fig. 10 E). In fact, treatment of *BA*^*tTA*^*:Gal3*^*−/−*^ HSCs with a GSK3β inhibitor restored aberrantly high nuclear β-catenin levels in leukemic HSCs (Fig. 10 F). Altogether, these results demonstrate that cytokines constitutively secreted by leukemic MPP3 can play a key role by locally enhancing myeloid lineage trajectory and amplifying myeloid cell production in the BM microenvironment, thereby contributing to disease progression. They also identify an important role for Gal3 in increasing Wnt activity in leukemic HSPCs, which could be therapeutically targeted to dampen the engagement of emergency myelopoiesis pathways in leukemic conditions.

## Discussion

How lineage specification occurs at the top of the hematopoietic hierarchy is a long-standing question in the field, with major implications for disease development and aging. Numerous studies have described the cellular and molecular heterogeneity of the HSC compartment and identified regulatory mechanisms underlying the differential production of lineage-biased MPPs (Olson et al., 2020; Yamashita et al., 2020). Here, we identified a new level of heterogeneity within the myeloid-biased MPP3 compartment, with immature FcγR^−^/ER^low^ MPP3 giving rise to all downstream myeloid progenitors, and secretory FcγR^+^/ER^high^ MPP3 quickly enhancing GMP and myeloid cell production through autocrine/paracrine signaling. This functional compartmentalization of the MPP3 population accounts for both its multipotent nature and myeloid-biased characteristics, with FcγR^+^/ER^high^ MPP3 representing a novel bypass mechanism for rapid and tunable production of myeloid cells. While evidence of lineage bypass mechanisms already exists in the literature, they are mostly based on re-evaluation of previous flow cytometry gating strategies (Paul et al., 2015; Pronk et al., 2007), interpretations of single cell transplantation experiments (Yamamoto et al., 2013), or analyses of lineage biases in reporter mice (Haas et al., 2018). Here, we provide one of the first cellular, molecular, and functional characterization of a myeloid bypass mechanism that starts in the MPP compartment, circumvents the controversial CMP stage of lineage decision, produces enough cytokines to drive the hematopoietic stress response, and kick-starts myeloid cell production by rapidly amplifying GMP production during emergency myelopoiesis.

A critical question arising when identifying a novel differentiation intermediate is whether it represents a functionally distinct population. Our results demonstrate that FcγR^+^/ER^high^ MPP3 are functionally distinct from the rare HSCs and truly multipotent FcγR^−^/ER^low^ MPP3, and from the much more abundant GMPs. While FcγR^+^/ER^high^ MPP3 and GMPs are clearly part of the same differentiation continuum and are both highly secretory following LPS/Pam3CSK4 stimulation, they are not equivalent cell populations having distinct surface markers, rough ER morphology, secretory patterns, molecular identity, and functionality. When tested in vitro, FcγR^+^/ER^high^ MPP3 are still capable of forming mixed colonies with residual megakaryocyte and erythroid lineage potential, which is totally lacking from GMPs and likely reflects their direct production from multipotent FcγR^−^/ER^low^ MPP3. Cytokines secreted by FcγR^+^/ER^high^ MPP3 are also much more effective at amplifying myeloid cell production from the HSPC compartment than GMP-secreted cytokines. However, in vivo, GMPs contribute to myeloid cell production in contrast to FcγR^+^/ER^high^ MPP3, which have no maintenance or expansion potential following infusion in recipient mice. This illustrates the short-lived transitional nature of FcγR^+^/ER^high^ MPP3 that is not compatible with a readout in transplantation assays, although it is well possible that they might persist much longer in undisturbed native conditions, as already shown for MPPs using in situ barcoding lineage tracking approaches (Sun et al., 2014; Busch et al., 2015; Rodriguez-Fraticelli et al., 2018). It also demonstrates that FcγR^+^/ER^high^ MPP3 function as an amplifying secretory compartment in the local BM niche that helps initiate emergency myelopoiesis. Considering the differences in cell numbers, it is likely that GMP-secreted cytokines will contribute to increased myeloid cell production from differentiating GMP clusters, while the much less abundant secretory MPP3 will assist in redirecting the differentiation potential of the upstream HSPC compartment toward myelopoiesis.

Our results identify MPP3 as one of the first myelopoiesis-regulatory populations that directly controls HSPC fate in their BM microenvironment. Here, we propose that the local secretion of pro-myeloid differentiation factors by FcγR^+^/ER^high^ MPP3 drives emergency myelopoiesis engagement in stress and disease conditions. The current lack of MPP3-specific genetic tools prevents us from directly testing the self-reinforcing role of this MPP3 subset, and from excluding the contribution of other secretory cells, but it is consistent with the emerging understanding of MPP biology as isolated cell types (Wu et al., 2022) that could help coordinate the response of the HSPC compartment and complement the effect of GMPs in amplifying myeloid cell production. In this context, it is likely that MPP3 will play an important role in amplifying myelopoiesis in other deregulated contexts such as infectious diseases and aging. Considering the autocrine and paracrine effects of MPP3-secreted cytokines in tailoring HSPC fate, it is also expected that MPP3 secretory function will underlie some of the cell-autonomous myeloid-biased HSC behaviors in single-cell transplantation experiments (Yamamoto et al., 2013; Haas et al., 2018). Furthermore, it is likely that other, still unknown, transitory states will act between the MPP compartment and GMPs to bridge other MPP populations like MPP2 and MPP4 to downstream progenitors and will serve specific, and likely distinct, functions at steady state and in emergency stress conditions. This might help redefine the identity of the controversial CMP compartment as a convergence of such transitory states.

Taken together, our findings identify a novel mechanism regulating myelopoiesis at the early stage of hematopoietic commitment, which represents an ideal cellular compartment to target therapeutically to rebalance lineage output in stress and disease conditions. With the current SARS-CoV-2 pandemic and the success of immunotherapy for cancer treatment, there is a well-justified interest in understanding the mechanisms controlling immune cell production particularly as it relates to innate immunity and myeloid cell production. Indeed, studies of “trained immunity” and the discovery of “central trained immunity” have demonstrated the importance of HSPCs and myeloid progenitors in the regulation of innate immune responses (Netea et al., 2020). Our identification of a myeloid bypass mechanism in the MPP3 compartment that produces enough cytokines to drive the hematopoietic stress response therefore warrants further preclinical studies and investigations in humans to understand its therapeutic potential for modulating myeloid cell production.

## Materials and methods

### Mice

All animal experiments were conducted at the University of California, San Francisco (UCSF) or Columbia University Irving Medical Center (CUIMC) in accordance with institutional animal care and use committee protocols approved at each institution, and in compliance with all relevant ethical regulations. Mice were bred and maintained in mouse facilities at UCSF or CUIMC in accordance with institutional animal care and use committee protocols approved at each institution. CD45.2 C57BL/6J (000664), CD45.1 C57BL/6-BoyJ (002014) and B6.Cg-*Lgals3*^*tm1Poi*^*/*J (006338) mice were purchased from the Jackson Laboratory. *Prdm1-Yfp* mice (Fooksman et al., 2010) were obtained from Dr. Mark Ansel (UCSF, San Francisco, CA, USA). *β-actin-Gfp*, *Scl-tTA:TRE-BCR/ABL*, and *Tnf*^−*/*−^ mice were previously described (Pietras et al., 2015; Reynaud et al., 2011; Yamashita and Passegué, 2019). *BA*^*tTA*^*:Gal3*^*−/−*^ mice were obtained by breeding B6.Cg-*Lgals3*^*tm1Poi*^/J mice with *Scl-tTA:TRE-BCR/ABL* mice. Respective WT littermates or single transgenic animals were used as Ctrl. 6- to 12-wk-old mice were used as a donor for cell isolation, and 8- to 12-wk-old congenic mice were used as recipients for transplantation experiments. For BCR/ABL induction, mice were withdrawn from doxycycline containing water at 5 wk of age. No specific randomization or blinding protocol was used with respect to the identity of experimental animals, and both male and female animals were used indiscriminately in all experiments. Animal facilities were maintained at 71 ± 2°F and 50 ± 10% relative humidity on a 12/12-h light/dark cycle. Mice were euthanized by CO_2_ asphyxiation followed by cervical dislocation.

### In vivo assays

For granulocyte depletion, mice were injected once intraperitoneally with 0.1 mg of IgG control (clone 2A3) or anti-Ly6G antibody (BP0075-1; BioXCell) in 200 µl PBS. For short-term in vivo lineage tracing assays, CD45.1 recipient mice were sublethally irradiated (8.5 Gy, delivered in split doses 3 h apart) using an x-ray irradiator (MultiRad225, Precision X-Ray Irradiation) and injected retroorbitally with 5,000 CD45.2 donor cells within the next 6 h. Irradiated recipient mice were administered polymyxin/neomycin-containing water for 4 wk following transplantation to prevent opportunistic infection and analyzed over time by repeated bleedings. Peripheral blood (PB) was obtained from retro-orbital plexus and collected in tubes containing 4 ml of 10 mM EDTA in ACK (150 mM NH_4_Cl/10 mM KHCO_3_) lysis buffer for flow cytometry analyses. For short-term in vivo differentiation assays, 10,000 donor cells isolated from *β-actin-Gfp* mice were retro-orbitally infused into recipient mice, which were analyzed for BM contribution at the indicated times.

### Flow cytometry

Staining of hematopoietic cells was performed as described previously (Kang et al., 2020). In brief, BM cells were obtained by crushing leg, arm, and pelvic bones in staining media composed of HBSS containing 2% heat-inactivated FBS (35-011-CV; Corning). RBCs were removed by lysis with ACK buffer, and single-cell suspensions of BM cells were purified on a Ficoll gradient (Histopaque 1119, Sigma-Aldrich). Spleens were mechanically dissociated in staining media and ACK lysed to remove contaminating RBCs. Blood was collected in ACK buffer containing 10% EDTA from intra-orbital bleed and further lysed in ACK buffer to remove contaminating RBCs. Cellularity was determined by ViCELL-XR automated cell counter (Beckman Coulter). For HSC and progenitor isolation, BM cells were pre-enriched for c-Kit^+^ cells using c-Kit microbeads (130-091-224; Miltenyi Biotec) and an AutoMACS cell separator (Miltenyi Biotec). Unfractionated or c-Kit-enriched BM cells were then incubated with purified rat anti-mouse lineage antibodies (CD3, 100202; BioLegend; CD4, 16-0041-82; eBioscience; CD5, 100602; BioLegend; CD8, 100702; BioLegend; CD11b, 101202; BioLegend; B220, 103202; BioLegend; Gr1, 14-5931-85; eBioscience; and Ter119, 116202; BioLegend) followed by goat anti-rat-PE-Cy5 (A10691; Invitrogen) and subsequently blocked with purified rat IgG (Sigma-Aldrich). Cells were then stained with Sca-1-PB (108120; BioLegend), c-Kit-APC-Cy7 (105826; BioLegend), CD48-A647 (103416; BioLegend), CD150-PE (115904; BioLegend), and Flk2-Bio (13-1351-85; eBioscience) followed by SA-BV605 (405229; BioLegend), CD34-FITC (11-0341-85; eBioscience) and FcγR-PE-Cy7 (101318; BioLegend). For ER-Tracker staining, BM cells were stained with 0.3 mM ER-Tracker green (E34251; Invitrogen) in Ca^2+^/Mg^2+^-containing HBSS (14025092; Gibco) for 15 min at 37°C in a 5% CO_2_ water jacket incubator. For plasma cell isolation, spleen cells from *Prdm1-Yfp* mice were stained with B220-APC-eFluor780 (47-0452-82; eBioscience) and CD138-APC (558626; BD Pharmingen). For in vitro myeloid differentiation of naïve HSCs, cultured cells were stained with Mac-1-FITC (11-0112-82; eBioscience) and FcγR-PerCP-eFluor710 (46-0161-82; eBioscience). For single-cell in vitro myeloid lineage differentiation assays, expanded clones were stained with CD45-APC-Cy7 (557659; BD Pharmingen), CD71-BUV395 (740223; BD Pharmingen), CD41-BV510 (133923; BioLegend), CD61-PE (104308; BioLegend), Gr-1-BV421 (108433; BioLegend), Mac-1-PE-Cy7 (25-0112-82; eBioscience), and FcεRI-APC (134316; BioLegend). For donor-derived chimerism analyses in transplanted mice, blood cells were stained with Gr-1-eFluor450 (48-5931-82; eBioscience), Mac-1-PE-Cy7, B220-APC-eFluor780, CD3-eFluor660 (50-0032-82; eBioscience), Ter-119-PE-Cy5 (15-5921-83; eBioscience), CD45.1-PE (12-0453-83; eBioscience), and CD45.2-FITC (11-0454-85; eBioscience). For short-term in vitro FcγR^−^ MPP3 and FcγR^+^ MPP3 culture, cells were stained with Sca-1-PB, c-Kit-APC-Cy7, CD48-A647, CD150-PE, Mac-1-FITC, and FcγR-PE-Cy7. For in vivo infusion of *β-actin-Gfp* cells, c-Kit-enriched recipient BM cells were stained with lineage cocktails as described above and then stained with Sca-1-PB, c-Kit-APC-Cy7, CD48-A647, CD150-PE, FcγR-PE-Cy7, and CD34-Bio (119304; BioLegend) followed by SA-BV605 (405229; BioLegend). Stained cells were finally resuspended in staining media containing 1 µg/ml propidium iodide for dead cell exclusion. Cell isolations were performed on a Becton Dickinson (BD) FACS Aria II (UCSF) or FACS Aria II SORP (CUIMC) using double sorting for purity. Cell analyses were performed on a BD LSR II (UCSF), BD Celesta (CUIMC), Agilent Novocyte Quanteon (CUIMC), or Bio-Rad ZE5 (CUIMC) cell analyzer. Data collection was performed using FACSDiva (v9) or Everest (v1) and analysis was performed in FlowJo (v9/v10).

### In vitro assays

All cultures were performed at 37°C in a 5% CO_2_ water jacket incubator (Thermo Fisher Scientific) and, except for TNFα, all cytokines were purchased from PeproTech. Mouse TNFα was obtained from Genentech under a material transfer agreement. To harvest supernatants, cells (10,000 per well of a 96-well plate) were grown in 150 µl base media consisting of IMDM (Gibco) with 5% FBS (StemCell Technology), 50 U/ml penicillin, 50 μg/ml streptomycin, 2 mM L-glutamine, 0.1 mM non-essential amino acids, 1 mM sodium pyruvate, and 50 μM 2-mercaptoethanol, and containing only stem cell factor (25 ng/ml), thrombopoietin (25 ng/ml), and Flt3-L (25 ng/ml) as cytokines. For stimulation, cells were cultured in the presence of 100 ng/ml LPS (L4391; Sigma-Aldrich) and 1 μg/ml Pam3CSK4 (4633; R&D Systems) for up to 24 h. For testing supernatant effects in liquid culture, naïve HSCs (1,000 cells per well of a 96-well plate) were grown in 150 µl of 100% harvested supernatants for either 6 d without any media replacement or 8 d with replenishing half of the culture supernatants every 3 d. For CFSE dilution assay, naïve cells (2,000 cells per well of a 96-well plate) were labeled with 2.5 μM CFSE (C1157; Molecular Probes) as described previously (Kang et al., 2020) and cultured for 3 d in 150 µl of 100% supernatant. For other liquid culture assays, cells (2,000–3,000 per well of a 96 well plate) were grown in 200 µl base media supplemented with IL-11 (25 ng/ml), IL-3 (10 ng/ml), GM-CSF (10 ng/ml) and erythropoietin (4 U/ml) for full cytokine cocktail, media and cultured for indicated time before analyses. Depending on experiments, different doses of IL-10 (210-10; PeproTech), 2 μM BMS-345541 (B9935; Sigma-Aldrich), 2 μM KN-93 (K1385; Sigma-Aldrich), and 30 µM CHIR 99021 (S1263; Selleckchem) were also added. To harvest supernatants for TNFα addition experiments, cells were exposed to 1 μg/ml TNFα (Genentech) in a full cytokine cocktail to ensure proper cell viability. For single-cell division counts, individual cells were directly sorted per well of a Terasaki plate containing 10 μl of full cytokine media, and the number of cells per well was manually counted under a microscope every 12 h. The single cell differentiation assay in liquid culture was adapted from a previously published protocol (McGrath et al., 2015). In brief, individual cells were directly sorted per well of a 96-well plate in 200 µl of IMDM containing 10% FBS (StemCell Technology), 20% BIT 9500 (9500; StemCell Technology), 5% PFHM II (12040077; Gibco), 50 U/ml penicillin, 50 μg/ml streptomycin, 2 mM L-glutamine, 0.1 mM non-essential amino acids, 1 mM sodium pyruvate, 55 μM 2-mercaptoethanol, stem cell factor (50 ng/ml), thrombopoietin (25 ng/ml), Flt3-L (10 ng/ml), IL-6 (10 ng/ml), IL-3 (10 ng/ml), GM-CSF (5 ng/ml), and erythropoietin (4 U/ml). Small megakaryocytic colonies were manually counted under a microscope on day 5, and all other colonies were harvested and scored by flow cytometry analyses after 6 d of culture. For methylcellulose colony assays, cells were plated into a 35-mm dish (100 cells/dish) containing 1 ml methylcellulose (M3231; StemCell Technologies) supplemented with 50 U/ml penicillin, 50 μg/ml streptomycin, and either the full cytokine cocktail described above or just 20% culture supernatants. Colonies were manually scored under a microscope after 8 d of culture.

### Cytokine analyses

Culture supernatants were clarified by filtering through 0.22-μm filter (SLGV004SL; Millipore) and stored at −80°C until use. BM fluids were prepared as previously described (Kang et al., 2020) and stored at −80°C until use. For ELISA measurements, 50 µl of culture supernatants were analyzed with IL-6 (50-172-18; eBioscience) or TNFα (88-7324-22; eBioscience) ELISA kits according to the manufacturer’s instructions. For Luminex cytokine multiplex bead arrays, 25 µl of culture supernatants were analyzed for a custom-made panel of five cytokines (IL-1β, IL-6, GM-CSF, TNFα, MIP1α; Invitrogen, PPX-05) according to the manufacturer’s instructions. For Raybiotech 200 mouse cytokine arrays, 500 µl of pooled culture supernatants or BM fluids were sent per sample to Raybiotech for quantitative proteomics services using Mouse Cytokine Array Q4000 kit. Quantile normalization was performed for direct comparison of secreted cytokine profiles from different array experiments using R Bioconductor package, and hierarchical clustering was conducted using pheatmap package of R (https://cran.r-project.org/web/packages/pheatmap/index.html). The statistical significance of the different distributions of secreted cytokine under diverse biological conditions was determined using Kruskal-Willis test (P value <0.05, R version 3.3: https://www.r-project.org).

### Single-cell secretion assays

Polydimethylsiloxane (PDMS) microwell array and antibody barcode glass slides were prepared and screened with FITC-labeled BSA as described (Chen et al., 2019), then blocked with 3% BSA for 1 h at room temperature (RT) and rinsed with base media just before usage. The PDMS microwell array was placed facing upward and the media was removed until just a thin layer remained on the array surface. Cell suspension (25,000 cells in 100 µl base media ± LPS/Pam3CSK4 stimulation) was pipetted onto the microwell array and allowed to settle for 10 min. The antibody glass slide was then put on top of the PDMS microwell array, with antibody barcode resting on the cell capture chambers, clamped tight together and imaged on an automatic microscope stage to acquire optical images recording the number and location of trapped cells in each microwell. Following imaging, the assembly was incubated for 18 h at 37°C in a 5% CO_2_ water jacket incubator, after which the antibody barcode glass slide was removed and submitted to ELISA immunoassay detection procedure. In brief, a mixture of biotinylated detection antibodies was pipetted onto the glass slide and incubated for 45 min at RT followed by washing with 3% BSA solution. APC dye-labeled streptavidin (17-4317-82; eBioscience) was added onto the glass slide and incubated for 30 min at RT, then washed with 3% BSA again and blocked with 3% BSA for 30 min at RT. The glass slide was then dipped sequentially in 2 Dulbecco’s PBS baths and 2 DI water baths before being finally blown dry. Genepix 4000B (UCSF) and 4200A (Yale) scanners (Molecular Devices) were used to obtain scanned fluorescent images for FITC (488 nm) and APC (635 nm) channels. Immunofluorescence and optical images were analyzed with GenePix Pro software (Molecular Devices) to align the microwells array template and extract fluorescence intensity values for wells determined to contain only a single cell. Fluorescence data were used to generate heatmap and scatterplots with Excel (Microsoft) and Prism (GraphPad).

### Immunofluorescence staining of cells

Isolated cells (2,000–3,000 in 5–10 µl of IMDM) were pipetted onto poly-L-lysine coated slides (P0425-72EA; Sigma-Aldrich), settled down for 15 min at RT, fixed with 4% paraformaldehyde for 10 min at RT, then washed three times with PBS, and permeabilized/blocked for 1 h at RT with 0.1% Tween-20 in 10% FBS (Corning) in PBS, which was then used as antibody incubation buffer for all the subsequent steps. Cells were incubated overnight at 4°C with a mouse monoclonal anti-KDEL (ab12223; Abcam) or a rabbit anti-mouse β-catenin (9582S; Cell Signaling) primary antibody, washed three times with PBS, and incubated for 1 h at RT with a goat anti-mouse IgG A488 (A11029; Invitrogen) or a donkey anti-rabbit-A555 (A31572; Invitrogen) secondary antibody. Cells were then washed three times with PBS, stained with 1 μg/ml DAPI (32670; Sigma-Aldrich) for 10 min at RT, washed three times with PBS, and finally slides were mounted with VectaShield (H-1000; Vector Laboratories). Cells were imaged on a Nikon Ti Eclipse inverted confocal microscope with 60× objective using Nikon NIS Elements (v3) for data collection, and images were processed using Fiji (https://fiji.sc). Cells were imaged on an Olympus epifluorescence microscope with 60× objective for manual scoring of nuclear β-catenin staining. At least 100 cells per condition were randomly captured for quantification.

### Immunofluorescence staining of bone sections

Femurs were embedded in OCT (Tissue-Tek, 4583), snap-frozen in a 100% ethanol/dry ice slurry, and kept at −80 °C until sectioning. Thin 7-μm sections were obtained upon cryosection at −30°C with a CryoJane tape transfer system (39475205; Leica) and a tungsten blade and were kept at −80°C before staining. Sections were first fixed with 100% acetone kept at −20°C for 10 min, dried for 5 min at RT, blocked for 90 min with 10% goat-serum (Gibco) in PBS, and washed three times with PBS for 5 min at RT. The same wash procedure was also used in between each staining step performed in 10% goat-serum in PBS. For HSPC staining, sections were incubated with A488-conjugated CD48 (103414; BioLegend) for 90 min at RT, blocked with 20 μg/ml Rat IgG (I8015-10MG; Sigma-Aldrich) for 10 min at RT, and then stained with PE-conjugated ESAM (136203; BioLegend) and Alexa647-conjugated lineage markers CD3e (100209; BioLegend), B220 (136203; BioLegend), Gr-1 (108418; BioLegend), Mac-1 (101218; BioLegend), and CD150 (115918; BioLegend) for 90 min at RT. For GMP staining, sections were incubated first with rat anti-mouse c-Kit (135102; BioLegend) primary antibody overnight at 4 °C in 10% goat-serum in PBS, followed by a goat anti-rat-Cy3 (112-165-167; Jackson ImmunoResearch) secondary antibody for 60 min at RT in 10% goat-serum in PBS and washed three times for 5 min with PBS at RT. Then, sections were stained with A488-conjugated lineage markers B220 (103225; BioLegend), Mac-1 (101217; BioLegend), Gr-1 (108417; BioLegend), CD3 (100210; BioLegend), Sca-1-A488 (108116; BioLegend), CD150-A488 (115916; BioLegend), and FcγR-A647 (101314; BioLegend) for 90 min at RT in 10% goat-serum in PBS. Sections were then counterstained with 1 μg/ml DAPI in PBS for 10 min at RT and mounted with Fluoromount G (0100-01; Southern Biotech) and imaged on an SP5 upright (UCSF) or SP8 inverted (CUIMC) confocal microscopes (Leica) with 20× objective using Leica Application Suite X (v3/v4) for data collection. Images were processed and analyzed using Volocity software (Perkin Elmer v.6.2), Imaris (Bitplane v.8.2), Photoshop (Adobe v.CS5), and Fiji (ImageJ2 v2.3). For quantification of the linear distance between HSPCs, the distance formula d = sqrt[(x_1_ − x_2_)^2^ + (y_1_ − y_2_)^2^] was used based on the (x,y) pixel coordinates of cells in the images. For MPP3 staining with the immunofluorescence scheme for in situ imaging, cells were stained by flow cytometry as described above and 1,000 MPP3 were directly deposited onto poly-L-lysine coated slides and fixed with 100% acetone for 5 min at −20°C. Of note, acetone fixation strips the flow cytometry surface markers allowing re-staining of isolated MPP3. Slides were then blocked for 90 min at RT with 10% goat serum in PBS, stained as described above for HSPC staining for tissue sections, and then mounted with VectaShield (H-1200; Vector Laboratories) containing 1 μg/ml DAPI and imaged on SP5 upright confocal microscope with oil immersion 63× objective. Images were processed using Volocity software, and 100 cells were scored to construct a library of representative images.

### Electron microscopy

Cells (50,000–100,000 per sample) were fixed in 0.1 M NaCacodylate (pH 7.4) containing 1% paraformaldehyde and 2% glutaraldehyde on ice for 30 min and pelleted at 3,000 ×*g* for 10 min at 4°C. Samples were then submitted to the Gladstone Institute (UCSF) Electron Microscopy Core Facility for standard TEM ultrastructural analyses. MPP3 images were manually scored for the presence or absence of cytoplasmic rough ER structures.

### SABiosciences UPR PCR array

Cells (5,000 per sample) were directly sorted into 500 μl Trizol LS (10296-010; Life Technologies) and RNA was extracted using Arcturus PicoPure RNA Isolation kit (KIT0204; Applied Biosystems) according to the manufacturer’s instructions. RNA concentration was measured using a bioanalyzer, and 1 ng of RNA was treated with DNase I (18068-015; Invitrogen) and reverse-transcribed using SuperScript III kit and random hexamers (18080-051; Invitrogen) to make cDNA. All cDNA samples were preamplified for pathway-specific genes and PCR arrays were performed according to the manufacturer’s instructions (PAMM-089Z; Qiagen). 7900HT Fast Real-Time PCR system was used to run the array, and the data were analyzed using the web-based SABiosciences RT^2^ Profiler PCR Array Data Analysis software. Values were normalized to *Gusb* expression.

### Bulk RNA-seq

Cells (5,000–7,000 per sample) were directly sorted into 350 μl RLT lysis buffer containing 1% β-mercaptoethanol from the RNeasy Plus Micro Kit (74034; Qiagen) and RNA was isolated according to the manufacturer’s instructions. RNA samples were submitted to the NYU Genome Technology Center for low-input RNA-seq. Briefly, RNA samples with RNA integrity number >9.5 as measured with a Bioanalyzer (Agilent Technologies) were subjected to further processing. Libraries were prepared using Trio RNA-seq Library Preparation Kit (NuGen, 0507) and sequenced on an Illumina NovaSeq 6000 for an average of ∼82.5 million paired reads per sample. The initial quality control of raw reads was performed using FastQC (https://www.bioinformatics.babraham.ac.uk/projects/fastqc/) for read quality assessment, and HISAT2 (Kim et al., 2015), the spliced junction mapper, to assess read duplication and read quality. The reference gene model of HISAT2 was based on the *Mus musculus* genome (GRCm38). The abundance of transcript reads was estimated using Salmon (Patro et al., 2017), and genes with low read counts were filtered out (<10 read counts across all samples). Differential gene expression analysis was carried out using R/Bioconductor DEseq2 package (Love et al., 2014). Normalized read counts were computed by dividing the raw read counts by size factors and fit to a negative binomial distribution. P values were first corrected by applying empirical estimation of the null distribution using the R package and then adjusted for multiple testing with the Benjamini–Hochberg correction. Genes with an adjusted P value <0.05 and fold change values >2 were considered differentially expressed. Volcano plots were generated using R (v.3.6.0) packages (ggplot2 and EnhancedVolcano). The enrichment of gene signatures based on KEGG pathway and GO term was examined using R package clusterProfiler (Yu et al., 2012) and IPA tool (Ingenuity Pathway Analysis, Qiagen). For HVG selection in K-means clustering analysis (Oyelade et al., 2016), the variance of the gene was quantified using the coefficient of variation (i.e., dispersion of expression over the mean of expression) of each gene expression across compared samples. To determine the statistical significance of HVGs, the background distribution of the coefficient of variation of gene expression was calculated using random permutation of gene expressions across samples. HVGs were then selected based on the calculated random distribution of the variance of gene expression (P value <0.05). Out of 19,639 genes, 981 genes were determined as HVGs of compared samples. With selected HVGs, K-means clustering approach identified the sequential pattern of gene expression changes across samples.

### Droplet-based scRNA-seq

For cultured samples, unfractionated MPP3 (50,000 cells per sample) and ER^high^/ER^low^ MPP3 subsets (30,000 cells per sample) were sorted per well of a 96-well plate into 150 µl base media ± LPS/Pam3CSK4 stimulation and cultured for 6 h. Cells were then washed and resuspended in HBSS/2% FBS at a concentration of 1,000 cells/ml. For freshly isolated samples, MPP3 (30,000–50,000 cells per sample) were directly sorted in HBSS/2% FBS at a concentration of 1,000 cells/ml. Samples were then submitted to the Columbia Genome Center Single Cell Analysis Core for microfluidic cell processing, library preparation, and sequencing. Briefly, cell viability and concentration were measured using a Countess II FL Automated Cell Counter (Thermo). RNA-seq library was prepared using a Chromium Single Cell 3′ Library & Gel Bead Kit v2 (10X Genomics) according to the manufacturer’s instructions. Samples (15,000 cells per sample per condition) were loaded and sequenced on an Illumina HiSeq4000 sequencer for an average of ∼326 million reads per sample. Sequencing data were aligned and quantified using the Cell Ranger Single-Cell Software Suite (version 2.0, 10X Genomics) against the *M. musculus* genome (GRCm38) provided by Cell Ranger. Using Seurat package of R (version 3; Butler et al., 2018), cells with fewer than 200 detected genes and of which the total mitochondrial gene expression exceeded 20% were removed. All of the raw read data passed the threshold of the proportion of doublet cells based on the DoubletFinder (<10% of doublet cells; McGinnis et al., 2019) and the number of gene expressions per cell (<7,500 expressions per cell). Bioinformatic analysis was conducted based on diverse R package and python-based analysis. The clustering of the preprocessed scRNA-seq data was based on the UMAP approach of the Seurat version3 package of R. The “FindAllMarkers” function of Seurat package was used to determine the list of cluster-defining genes. To evaluate biological signatures associated with a specific cell type within MPP3, we calculated gene module scores for each cell type using “AddModuleScore” function of Seurat (Tirosh et al., 2016). To establish gene signatures for this module scoring approach, the “FindConservedMarkers” function of Seurat was used to determine conserved gene lists for HSC and GMP from three independent LK and LSK scRNA-seq datasets from WT, adult C57BL/6 mice (unpublished data). The “CellCycleScoring” function was used to infer the cell cycle status of each cell based on regression approach and the “FeaturePlot” function was used to highlight a group of cells of interest in the UMAP representations. For comprehensive integration and comparison of scRNA-seq data across samples from diverse experimental conditions, nearest-neighbor integration was used. We used GO (Huang da et al., 2009) for pathway analyses and inferred the trajectory of cell state transitions using Slingshot (Street et al., 2018) of the R package based on the analysis results of Seurat v3. The RNA velocity of single cells, which is a high-dimensional vector that predicts the future state of individual cells, was inferred by the python package scVelo (La Manno et al., 2018) using the dynamic model with unfixed RNA kinetic parameters. Due to the use of pooled MPP3 cells from 10 to 12 mice, we utilized a broad set of genes, which was shared by more than 50 cells to involve a spectrum of steady-state RNA velocity covering multiple samples. Based on the inferred velocity of a single cell, we determined the directionality of the presented trajectory of cell state transitions in the Slingshot analysis.

### Quantification and statistical analysis

All experiments were repeated as indicated; *n* indicates the number of independent biological repeats. Data are expressed as mean ± SD. Mice for treatment and transplantation were randomized, samples were alternated whenever possible, and no blinding protocol was used. Statistical significance was evaluated by a two-tailed unpaired Student’s *t* test unless otherwise indicated. P values <0.05 were considered statistically significant. Figures were made with GraphPad Prism software.

### Online supplemental material

Fig. S1 details the secretory activity of HSPCs. Fig. S2 shows the autocrine effect of MPP3 secretion at a single-cell level. Fig. S3 expands on the molecular changes of MPP3 upon inflammatory stimulus. Fig. S4 details the molecular rewiring and constitutive cytokine secretion of leukemic MPP3. Fig. S5 shows the changes in the leukemic BM niche microenvironment. Table S1 lists the cytokines uniquely or commonly secreted by HSCs, MPP3, MPP4, and GMPs with or without stimulation. Table S2 lists HSC and GMP signature genes. Table S3 details the scRNA-seq analyses of MPP3 subsets. Table S4 lists the DEGs between ER^high^ and ER^low^ MPP3 subsets and GMPs. Table S5 details leukemic *BA*^*tTA*^ MPP3 and *BA*^*tTA*^ mice secretomes.

## Supplementary Material

Table S1 shows the representative HSPC secretome.

Table S2 lists HSC and GMP signature genes.

Table S3 provides analyses of MPP3 scRNA-seq clusters.

Table S4 lists significantly differentially expressed genes.

Table S5 lists leukemic *BA*^*tTA*^ MPP3 and *BA*^*tTA*^ mice secretomes.

## Data Availability

RNA-seq data have been deposited in the Gene Expression Omnibus under accession code GSE181902. Source data for all the figures are provided in the paper. All other data are available from the corresponding author upon reasonable request. All code and packages used to support the findings of this study are either publicly available or available from the corresponding author upon reasonable request.
